# A Many-Objective Evolutionary Algorithm Based on Dual Selection Strategy

**DOI:** 10.3390/e25071015

**Published:** 2023-07-01

**Authors:** Cheng Peng, Cai Dai, Xingsi Xue

**Affiliations:** 1 School of Computer Science, Shaanxi Normal University, Xi’an 710119, China; pc@snnu.edu.cn; 2 Fujian Provincial Key Laboratory of Big Data Mining and Applications, Fujian University of Technology, Fuzhou 350118, China; jack8375@gmail.com

**Keywords:** many-objective optimization, convergence, diversity, dual selection

## Abstract

In high-dimensional space, most multi-objective optimization algorithms encounter difficulties in solving many-objective optimization problems because they cannot balance convergence and diversity. As the number of objectives increases, the non-dominated solutions become difficult to distinguish while challenging the assessment of diversity in high-dimensional objective space. To reduce selection pressure and improve diversity, this article proposes a many-objective evolutionary algorithm based on dual selection strategy (MaOEA/DS). First, a new distance function is designed as an effective distance metric. Then, based distance function, a point crowding-degree (PC) strategy, is proposed to further enhance the algorithm’s ability to distinguish superior solutions in population. Finally, a dual selection strategy is proposed. In the first selection, the individuals with the best convergence are selected from the top few individuals with good diversity in the population, focusing on population convergence. In the second selection, the PC strategy is used to further select individuals with larger crowding distance values, emphasizing population diversity. To extensively evaluate the performance of the algorithm, this paper compares the proposed algorithm with several state-of-the-art algorithms. The experimental results show that MaOEA/DS outperforms other comparison algorithms in overall performance, indicating the effectiveness of the proposed algorithm.

## 1. Introduction

Evolutionary algorithms are widely used to optimize various problems [1,2], and one of the important applications is multi-objective optimization problems (MOPs), which exist in many practical engineering problems [3,4,5,6,7]. In many conflicting problems, we need to consider optimizing multiple objectives simultaneously, and these objectives often conflict with each other. The following are some applications of multi-objective optimization in practical engineering. In truck cab design [3], a multi-objective particle swarm optimization algorithm is used to make a proper tradeoff between the lightweight and fatigue durability for the design of the truck cab. The maximization of fatigue life and minimization of lightweight are chosen as two competing objectives to be optimized within a multi-objective framework. One example of feature selection [4] proposed an algorithm for feature selection of high-dimensional data, where the three objectives’ number of features, balanced classification error rate, and distance metric are considered simultaneously, with the goal to minimize these three objectives. In food recommendation problems [5], the original recommendation problem is transformed into a four-objective mathematical model and many-objective optimization algorithms are used to optimize and maximize the four objectives: user preferences, numerical values, food diversity, and dynamic time wrapping. A multi-objective fuzzy decision-making model [6] is proposed for coal production, which includes five objectives: maximizing economics, guiding economics, safety investment, minimizing energy, and environment. In article  [7], the many-objective genetic algorithm is used to solve the example of supply chain, with the goal to minimize the six objectives: order cost, holding cost, transportation cost, main warehouse shortage cost, overflow cost, and support warehouses shortage cost.

In general, the minimum MOP is defined as:

(1) $$\left\{\begin{array}{c} \min F\left(X\right)=\left(f_{1}\left(X\right),f_{2}\left(X\right),\ldots ,f_{M}\left(X\right)\right)\\ \;\mathrm{subject}\;\mathrm{to}\;X\in R^{D} \end{array}\right.$$

where *M* represents the number of objectives; $X=(x_{1},x_{2},\ldots ,x_{D})$ is the *D*-dimensional decision vector; $R^{D}$ represents the *D*-dimensional decision space; and F(X) contains *M* objective functions $F=[f_{1},f_{2}\ldots ,f_{M}]$. When the number of objectives of the MOP is *M* ≥ 4, it is called the many-objective optimization problem (MaOP). Compared to single-objective problems, the objectives in many-objective problems conflict with each other, which means that it is impossible to have an optimal solution to achieve the best performance of all objectives. Therefore, the method of solving multi-objective problems is to find a set of solutions that can be a compromise for all objectives. In the decision space, for any two solutions *x* and *y*, if they satisfy ∀i∈1,⋯,M, $f_{i}\left(x\right)\leq f_{i}\left(y\right)$ and ∃i∈1,…,M, $f_{i}\left(x\right)<f_{i}\left(y\right)$, we say *x* dominates *y* or *y* is dominated by *x*. The solution that is not dominated by any solution is defined as a Pareto optimal solution. The Pareto set (PS) consists of all Pareto optimal solutions, while the objectives corresponding to the solutions in the PS constitute the Pareto front (PF).

In the past few decades, a large number of multi-objective evolutionary algorithms (MOEAs) have been proposed. MOEAs aim to obtain a solution set with both convergence and diversity. So far, according to the selection mechanism, the methods to solve MOPs or MaOPs can be divided into four categories.

Pareto dominance-based algorithms belong to the first category. They enhance the selection pressure of the algorithm through improved or relaxed dominance relation. NSGAII [8] is a representative algorithm of this category and is widely used [9]. Later, scholars proposed some other dominance relations. For example, ε-dominance  [10], α-dominance [11], fuzzy dominance [12], etc. In article [13], a method based on grid domination is proposed, which enhances the selection pressure through three grid-based criteria. Zhang et al.  [14] adopted the knee point-based selection scheme to select non-dominated solutions. Although the above algorithm can accelerate the convergence of the population, the improved dominance relationship will lead to the deterioration of the diversity of the population [15]. At present, several new dominance relations have been developed, such as SDR [16], CSDR [17], and MultiGPO [18].

Indicator-based algorithms form the second category. These MAOEs use indicators to guide population evolution. Hypervolume (HV) [19,20] is a strictly Pareto-compliant and widely used indicator, but the time to calculate HV is expensive, so HypE [21] uses Monte Carlo simulation to approximate the HV value of the solution. Inverted generational distance (IGD) [22] is also a popular index to measure the convergence and diversity of the solution set. The hypervolume adaptive grid algorithm (HAGA) [23] only calculates the contribution hypervolume index of the grid population, which relatively saves the calculation time. In addition, other indicators include ΔP [24], pure diversity (PD) [25], and coverage PF (CPF) [26]. However, these methods have high computational complexity.

Decomposition-based algorithms are the third category. The main idea is to decompose the MaOP into several sub-problems and solve them one by one, with the MOEA/D [27] algorithm being a representative algorithm of this kind that is widely used [28]. NSGA-III [29] uses Pareto dominance to emphasize convergence and uses uniformly distributed reference points to manage diversity. MOEA/D-M2M [30] collaboration solves the sub-problems. RVEA [31] is a reference vector-guided EA, which uses a scalarization method to balance the convergence and diversity of solutions. However, the performance of these methods on different problems is uncertain, and the effect is poor when dealing with irregular PFs.

Environmental-based selection is the fourth category, and the selection process can be roughly divided into two steps. In [32], the first step is to select only one solution with the best convergence indicator. In the second step, the diversity is measured according to the cosine similarity, and the solution close to the first step is further selected. In [33], the first step is to use the achievement scalarization function based on the R2 indicator for primary selection. The second step takes advantage of the reference vector-guided objective space partition approach in diversity management for many-objective optimization. In [34], in the first step, a solution with a small neighborhood density is selected to form a candidate pool, where the neighborhood density of the solution is calculated based on a new adaptive position transformation strategy. In the second step, the best solution for convergence from the candidate pool is selected and inserted into the next generation.

Although the existing methods have made significant progress in solving MaOPs, there are still many challenges. First, as the number of objectives *M* increases, there is a dominance resistance phenomenon [35] where the number of non-dominated solutions increases exponentially. Secondly, due to the curse of high-dimensionality, the evaluation of diversity becomes difficult [31]. In addition, many important problems such as high computational complexity and difficulty in balancing convergence and diversity need to be solved [36].

It is well known that convergence and diversity are two key factors in the performance of the MaOEA, which play different roles at different stages of the evolutionary process. Specifically, the solution may not converge in the early search stage, so more convergence pressure is needed to accelerate convergence. In the later search stage, the solution set is basically close to PF. At this time, it is necessary to eliminate crowding and select a better distributed solution set (i.e., good diversity). Therefore, we divide the whole selection process into two stages, which is also inspired by the fourth type of environment selection method. A dual selection strategy is proposed to avoid the negative impact of potential conflicts between convergence and diversity. Specifically, in the first selection, convergence is emphasized on the basis of diversity, and then diversity is emphasized with the proposed PC strategy in the second selection. The main contributions of this article are summarized as follows:

(1) This article proposes a dual selection strategy. In the first selection, the individuals with the best convergence are selected from the top few individuals with good diversity in the population, focusing on population convergence. In the second selection, the proposed PC strategy is used to further select individuals with large crowding-degree values, emphasizing population diversity.

(2) A new distance function is designed as a more effective distance metric. In addition, when evaluating the diversity of the solution set, the PC strategy based on new distance function not only considers the distance between the nearest two points in the objective space as large as possible, but also considers the difference between each objective function as large as possible and considers the influence of multiple points around on the diversity of this point, so as to obtain a better diversity of the solution set.

(3) Extensive comparisons are made between MaOEA/DS and several state-of-the-art algorithms for 93 instances of 31 test problems from 3 well-known test suites. The results show that MaOEA/AS is a promising MaOEA.

The remainder of this article is organized as follows. Section 2 introduces the proposed algorithm in detail. In Section 3, the experimental results and correlation analysis of the algorithm are given. Finally, the conclusion is given in Section 4.

## 2. Proposed Algorithm

### 2.1. Framework of MaOEA/DS

Algorithm 1 describes the main framework of the proposed algorithm, MaOEA/DS. First, a population *O* of size *N* is randomly generated. Then, more potential solutions $O^{\prime }$ in the solution pool are selected. Next, the reproduction operation is used to generate offspring $O^{\prime \prime }$ using simulated binary crossover and polynomial mutation. Then, $O^{\prime }$ and $O^{\prime \prime }$ are combined to generate a new population *R*. Finally, dual selection is performed to select the best performance individual from *R*. This evolutionary search process is repeated until the stop condition is satisfied, which finally outputs the final population. In addition to improvements in ChooseSolutions and environment selection, the basic process of the algorithm is similar to most other MOEAs. Other key components of MaOEA/DS are described in detail below.
**Algorithm 1** The framework of the algorithm for MaOEA/DS**Require:** 
*N* (population size)**Ensure:** 
*O* (final population)1:*O* = Initialization (*N*)2:While the stopping criterion is not met do3:   $O^{\prime }$ = ChooseSolutions (*O*)4:   $O^{\prime \prime }$ = Reproduction ($O^{\prime }$)5:   $R=O^{\prime }\cup O^{\prime \prime }$6:   *O* = DualSelection (R,N)7:Endwhile8:**return** *O*;

### 2.2. Convergence Measurement

Population convergence is a pivotal problem in MaOEA design. One of the simplest ways to measure convergence is to sum the fitness values of each objective. In this paper, we use the achievement scalarization function (ASF) to measure the convergence of each solution [29]. The ASF has been widely used to measure the convergence of each solution. The smaller the ASF value of the solution, the better the convergence performance. Assuming that there are *N* solutions, expressed as $x_{j}=1,2,\ldots ,N$, then the definition of the ASF is as follows:

(2) $$\begin{array}{c} I_{c}\left(x_{j}\right)=\mathrm{ASF}\left(x_{j},z_{i}^{\min},w\right)=\max_{i=1}^{M}\left(\frac{f_{i}\left(x_{j}\right)-z_{i}^{\min}}{w_{i}}\right) \end{array}$$

where $z_{i}^{\min}$ is the minimum value of each objective of all solutions in *N*, *M* is the number of objectives, *w* is the weight vector, and the weight vector of the i-th element is defined as:

(3) $$\begin{array}{c} w_{i}=\frac{f_{i}\left(x_{j}\right)}{\sum_{k=1}^{M}f_{k}\left(x_{j}\right)}. \end{array}$$

Note here that if $w_{i}$ is equal to 0, it is set to $10^{-6}$.

### 2.3. Diversity Measurement

#### 2.3.1. Calculation Diversity

It is very important to measure the diversity of the population effectively in the whole evolution process. However, with the rapid increase in the number of objectives, some of these methods are not suitable for high-dimensional objectives or cannot handle the problem of the PF with different shapes, for example, Euclidean distance and Manhattan distance. In order to solve these problems, this article proposes a new distance function to measure, which not only considers the distance between the nearest two points in the objective space, but also considers the difference between each objective function when evaluating the diversity of the solution set. The specific function is as follows. Assuming that the set of the current objective space is $A=\{x^{1},\cdots ,x^{n}\}$, $x^{i}=\left(x_{1}^{i},\cdots ,x_{M}^{i}\right)$, we first calculate the distance between $x^{i}$ and $x^{j}$ at any two points:

(4) $$\begin{array}{c} I_{d}\left(x^{i},x^{j}\right)=d\left(x^{i},x^{j}\right)={∥x^{i}-x^{j}∥}_{2}+\theta *\prod_{\left(k=1\right)}^{M}\sqrt{1-{\left(\frac{|x_{k}^{i}-x_{k}^{j}|}{∥x^{i}-x^{j}{∥}_{2}}\right)}^{2}} \end{array}$$

where Θ is a parameter greater than 0 and $\sqrt{1-{\left(\frac{|x_{k}^{i}-x_{k}^{j}|}{∥x^{i}-x^{j}{∥}_{2}}\right)}^{2}}$ is the sine value of the angle between the straight line $x^{i}-x^{j}$ and $L=\left(L_{1},\cdots ,L_{M}\right)(L_{k}=1,\;$ other components are 0). All these sine values are multiplied so that the included angle between $x^{i}-x^{j}$ and $L=\left(L_{1},\cdots ,L_{M}\right)(L_{k}=1,\;$ other components are 0) is as small as possible ($|x_{k}^{i}-x_{k}^{j}|$ as large as possible), which ensures that each component will be as different as possible. A good set of diversity is when the area it dominates is as large as possible. In other words, large objective space, less sampling points. Therefore, considering the differences between each objective function, we try to have a relatively large difference between the components of any two points. For example, the first dimensional component of the first point is 0.5, and the difference between the first components of other points and 0.5 should be as large as possible.

#### 2.3.2. PC Strategy

The method of evaluating the diversity of the solution set can directly affect the final performance of the algorithm. The point crowding degree is a widely used method for maintaining diversity in the field of multi-objective optimization. Crowding degree can help us maintain diversity and good distribution when selecting solution sets. By calculating the crowding degree of each solution, the density of the entire solution set can be determined, and the PF can be selected according to the crowding degree. Because some solutions may be closer to the PF, while others may be sparser or scattered, we can choose to have higher point crowding degree values to ensure the selection of solutions with good diversity.

The calculation method of point crowding degree usually involves dividing the objective function space into discrete grids and calculating the density and distance of the solutions in each grid. Euclidean distance or Manhattan distance is usually used to calculate the distance difference between solutions. Aiming at the characteristics of high dimensional many-objective evolutionary problems with high dimension and complexity, based on a new distance function, this article proposes a new PC strategy. The calculation process of the PC strategy is as follows. Then, for any point $x^{j}$, we determine its neighbors by the following:

(5) $$\begin{array}{c} \begin{array}{cc} & H=\{x|x_{k}=min\left\{t|t\geq x_{k}^{j},t\in \left\{x_{k}^{1},\cdots ,x_{k}^{n}\right\}\right\},or\;\\ & x_{k}=max\left\{t\leq x_{k}^{j},t\in \left\{x_{k}^{1},\cdots ,x_{k}^{n}\right\}\right\},\\ & x\;=(x_{1},\cdots ,x_{M})\in A,k=1,\cdots ,M,x\neq t\} \end{array} \end{array}$$

Then, we calculate the crowding degree of $x^{j}$:

(6) $$\begin{array}{c} CD\left(x^{j}\right)=\sum_{\left(y\in H\right)}d\left(x^{j},y\right)/\left|H\right| \end{array}$$

where $d\left(x^{j},y\right)$ is calculated by Equation (4) and |H| represents the size of the set *H*. The crowding degree $CD(x^{j})$ of a point not only considers the Euclidean distance between two points, but also considers the difference between each component, and finally considers the influence of the neighbor solution on its crowding degree. The greater the $CD(x^{j})$ value, the better the sparsity of $x^{j}$. By calculating the crowding degree of each individual, we can delete the individuals with the smaller crowding degree value and then maintain the diversity of the population. The core of the PC strategy is to determine the neighbors of each point and measure the distance between two points to achieve our goal (the difference between any components should be as large as possible). Therefore, ultimately, individuals with a high crowding degree will be selected for retention.
**Algorithm 2** PC strategy**Require:** 
$O_{T}$ (temporary population), *N* (population size)**Ensure:** 
*O* (final population)1:While |O| < *N*2:   For each solution in $O_{T}$ do3:      Randomly select an individual to determine its neighbors by Equation (5)4:      Calculate the crowding degree value of individual by Equation (6)5:   End6:   Put the individual *o*=arg max(CD(x)) to *O*7:   O=O∪o8:   Update $O_{T}$ and remove *o* from $O_{T}$9:Endwhile10:**return** *O*

### 2.4. ChooseSolutions

ChooseSolutions is very vital for the whole evolution. Individuals with good convergence or diversity are selected as parents to guide the search. Algorithm 3 shows the process of ChooseSolutions. In order to better introduce stronger selection pressure and maintain better diversity, we use the ASF and new distance function to calculate $I_{c}$ and $I_{d}$, respectively. Algorithm 3 shows more details of choosing solutions. First, two individuals are randomly selected from *O*. Comparing the metrics of the two individuals, the individual with smaller $I_{c}$ value and larger $I_{d}$ value enter the solution pool. Otherwise, an individual will be randomly selected to enter the solution pool. The process continues until the solution pool *M* is full.
**Algorithm 3** ChooseSolutions**Require:** 
*O* (initial population), *N* (population size)**Ensure:** 
$O^{\prime }$ (parent population)1:*O* = *⌀*2:While $|O^{\prime }|$ < *N* do3:   Randomly select two solutions $p_{1}$ and $p_{2}$ from population *O*4:   If $I_{c}$($p_{1}$) <$I_{c}$($p_{2}$) & $I_{d}$($p_{1},o$) >$I_{d}$($p_{2},o$)(o∈O,o≠p1,p2)5:      $O^{\prime }=O^{\prime }\cup p_{1}$6:   ElseIf $I_{c}$($p_{1}$)>$I_{c}$($p_{2}$) & $I_{d}$($p_{1},o$) <$I_{d}$($p_{2},o$)(o∈O,o≠p1,p2)7:      $O^{\prime }=O^{\prime }\cup p_{2}$8:   Else9:   Select one randomly10:   End11:EndWhile12:**return** $O^{\prime }$;

### 2.5. Dual Selection

Execution environment selection aims to select the best performance individual from the union set, where the convergence and diversity of the balanced solution set are crucial. Algorithm 4 shows the details of the dual selection procedure. Firstly, the union set *R* is normalized in the objective space. Then, the $I_{c}$ values of all individuals in *R* are calculated. According to Equation (2), the solution of the minimum $I_{c}$ value is found and substituted into the temporary optimal set $O_{T}$. All individuals in *R* are non-dominated and sorted to form a non-dominated set $R_{N}$. Next, we execute the dual selection strategy.

In the first selection (lines 6–12), the $I_{d}$ value from the non-dominated individuals in *R* to the individuals in $O_{T}$ is calculated by Equation (4). Corner individual refers to the individual at the edge of the Pareto front solution set. Then, the $I_{d}$ value of corner individuals is set to +*∞* because they are the most representative individuals on the PF and will definitely be selected into $O_{T}$. All individuals are sorted in descending order according to the $I_{d}$ value. Then, the individual *s* with the minimum $I_{c}$ value in the top *N*-$|O_{T}|$ individuals is found, i.e., the one with the best convergence. This process enhances the convergence pressure on the basis of maintaining diversity, and repeats the operation until the population reaches the condition.

In the second selection (lines 13–14), firstly, the temporary optimal set $O_{T}$ is combined with the non-dominated set $R_{N}$. In this process, we mainly evaluate the diversity performance of the solutions. We use a new PC strategy, whose details are shown in Algorithm 2. First, each solution in $O_{T}$ performs the following two steps: each individual determines its neighbors according to Equation (5), and then determines its crowding degree according to Equation (6). The individual with the largest crowding degree in all candidate solutions is selected in turn, and then it is removed from the candidate solution to update the candidate set. Next, we end the dual selection and, finally, output *N* individuals with the best performance. In general, the first selection improves the convergence of the solution set on the basis of maintaining diversity, the second selection selects individuals with better distribution, and the dual selection balances the convergence and diversity of the solution set.
**Algorithm 4** Dual selection**Require:** 
*R* (combined population), *N* (population size)**Ensure:** 
*O* (final population)1:$O_{T}$ = *⌀*2:*R*=Objective space Initialization(*R*)3:Calculate convergence value $I_{c}$ of all individuals in *R* by Equation (2)4:Put the individual $s=argmin(I_{c})$ to $O_{T}$5:Non-dominated sorting of all individuals in *R* and get a set $R_{N}$6:While $|O_{T}|$ < *N* do7:   Calculate diversity value $I_{d_{x^{i}\in R}}=\max_{x^{j}\in O_{T}}d\left(x^{i},x^{j}\right)$ by Equation (4)8:   Set the $I_{d}$ value of each corner individual to +*∞*9:   Descending order according to $I_{d_{x^{i}\in R}}$ value of all individuals10:   Find *s*= arg min($I_{c}$) in top ranked *N*-$|O_{T}|$ individuals11:   Remove *s* from *R*12:EndWhile13:$O_{T}=O_{T}\cup$$R_{N}$14:*O* = PC strategy ($O_{T}$)15:**return** (*O*);

### 2.6. Computational Complexity Analysis

In this section, assuming that the number of objectives is *M* and the population size is *N*, we analyze the time complexity of MaOEA/DS per generation. In ChooseSolutions, the calculation of the ASF value and $I_{d}$ distance value requires O(MN) and O($MN^{2}$), and the complexity of double selection is O ($MN^{2}$), so the time complexity of MaOEA/DS is O($MN^{2}$).

## 3. Experimental Research

To verify the performance of MaOEA/DS, we compare it with five state-of-the-art many-objective evolutionary algorithms, NSGAIII [29], MOEA/DD [37], onebyoneEA [32], SPEAR [38], and RVEA [31], on the widely used MaF, WFG, and DTLZ test suites. NSGA-III [29] supplies and updates well-spread reference points adaptively to maintain the diversity among population members. MOEA/DD [37] combines dominance- and decomposition-based approaches to balance the convergence and diversity of the evolutionary process. onebyoneEA [32] selects the solutions one by one. The first step is to select a solution with good convergence, and the second step is to select a solution with good diversity. SPEAR [38] introduces an efficient reference direction-based density estimator, a new fitness assignment scheme, and a new environmental selection strategy for handling MaOPs. RVEA [31] adopts a scalarization approach named angle-penalized distance to balance convergence and diversity.

### 3.1. Experimental Settings

In this article, MaF1-15, WFG1-9, and DTLZ1-7 are selected for experimental comparison at 5, 10, and 15 objectives. Table 1 gives the parameter settings and characteristics of these problems, where *M* is the number of objectives and *D* is the number of decision variables. Each algorithm runs independently 20 times on each test problem. Finally, the results of the proposed MaOEA/DS algorithm and five comparison algorithms are analyzed using the Wilcoxon rank sum test with a significance level of 0.05. In this experiment, the θ in the PC strategy is 0.5. For a fair comparison, in the reference [18], under the same *M*, we set the population size (represented as N) of all algorithms to be the same, with 210, 275, and 240 for 5, 10, and 15 objectives, respectively. The maximum generation (Gmax) is adopted as the termination criterion for all algorithms, which is set to 200 for the WFG problems and 500 for the MaF problems. The number of maximum fitness evaluation (MaxFE) = Gmax ∗ N. These algorithms are implemented on a PC equipped with Intel (R) Core (TM) i5-7500 CPU @ 3.40 GHz 3.41 GHz (Windows 10 operating system) using MATLAB language.

### 3.2. Performance Metrics

This article adopts Inverted Generational Distance (IGD) and Pure distance (PD) indicators to measure the comprehensive performance and diversity of different algorithms on various test problems. The advantage of IGD is its computational efficiency and versatility, which can simultaneously measure the convergence and diversity of solutions. IGD [39] can be calculated by:

(7) $$\begin{array}{c} IGD\left(S,S^{*}\right)=\frac{\sum_{x\in S^{*}}min_{y\in S}dist\left(x,y\right))}{|S^{*}|} \end{array}$$

where *S* is the solution set obtained by the algorithm, $S^{*}$ is composed of evenly distributed reference points sampled from the true PF, and dist(x,y) denotes the Euclidean distance between solution *y* in *S* and solution *x* in $S^{*}$. IGD measures the average minimum distance from each solution from $S^{*}$ to *S*. For an algorithm, a smaller IGD value means a better quality of the objective vectors of obtained solutions for approximating the PF.

PD [25] proposed by Wang et al., should measure diversity by calculating the dissimilarity between solution *x* and solution set *S* for MaOPs, which is defined as follows:

(8) $$\begin{array}{c} PD\left(S\right)=\max_{x^{i}\in S}\left(PD\left(s-x^{i}\right)+d\left(x^{i},S-x^{i}\right)\right) \end{array}$$

the calculation of *d* is as follows:

(9) $$\begin{array}{c} d\left(x,S\right)=\min_{x^{i}\in S}\left(\mathrm{dissimilarity}\left(x,x^{i}\right)\right) \end{array}$$

the calculation process of dissimilarity can be referenced [25], and the larger the PD, the better the diversity of the solution set.

### 3.3. Experimental Results and Analysis

In this article, the proposed MaOEA/DS algorithm is compared with 5 other algorithms on 45 MaF test problems and 27 WFG test problems. For each test problem, the result with the best performance is marked in bold. “+” means that MaOEA/DS is worse than its competitor algorithm, “−” means that MaOEA/DS outperforms its competitor algorithm, and “=” means that the competitor algorithm has the same performance as MaOEA/DS.

#### 3.3.1. MaF Suite

Table 2 reports the IGD mean and standard deviation values obtained by 6 MOEAs on 45 MaF test problems. Of the 45 problems, the statistical performance of MaOEA/DS on 26 problems is better than that of the comparison algorithm, which shows the good performance of the algorithm in IGD form. NSGAIII, MOEA/DD, onebyoneEA, SPEAR, and RVEA outperform MaOEA/DS on 11, 8, 11, 8, and 16 problems, respectively, while MaOEA/DS outperforms NSGAIII, MOEA/DD, onebyoneEA, SPEAR, and RVEA on 32, 31, 31, 35, and 27 problems, respectively.

For 8 problems (MaF1, MaF2, MaF4, MaF5, MaF7, MaF8, MaF9, and MaF15) of the partial PFs with incomplete coverage of the unit hyperplane, the IGD mean values of the MaOEA/DS algorithm on 18, 19, 18, 20, and 16 problems are smaller than those of NSGAIII, MOEA /DD, onebyoneEA, SPEAR, and RVEA algorithms, indicating that the MaOEA /DS algorithm has the best overall performance in IGD form on most problems. For the PF degradation problem MaF6, MaOEA/DS performs better than NSGAIII, MOEA/DD, onebyoneEA, SPEAR, and RVEA on 3, 3, 2, 3, and 3 problems.

When dealing with the problem of 6 PF projections completely covering the unit hyperplane (MaF3, MaF10, MaF11, MaF12, MaF13, and MaF14), the IGD mean obtained by the MaOEA/DS algorithm is smaller than the IGD mean obtained by the NSGAIII, MOEA/DD, onebyoneEA, SPEAR, and RVEA algorithms on 11,9,11,12, and 8 problems.

Table 3 reports the PD mean and standard deviation values obtained by 5 MOEAs on 45 MaF test problems. Of the 45 problems, the statistical performance of MaOEA/DS is better than that of the comparison algorithm on 23 problems. MaOEA/DS performs better than NSGAIII, MOEA/DD, onebyoneEA, and RVEA on 27, 36, 37, and 35 problems, and shows significant advantages in MaF1, MaF2, MaF10, MaF11, and MaF12.

**Figure 1:**
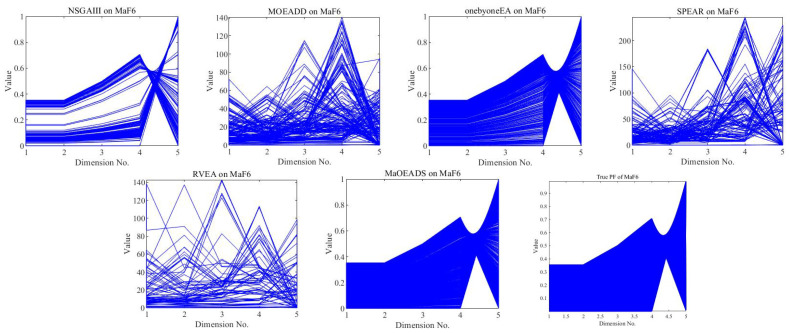
The final solution set obtained by the six MOEAs on five-objective MaF6.

Next, we more intuitively observe the ability of the six algorithms to balance convergence and diversity on the MaF test suite. Because scatter plots may only be drawn readily in 2D or 3D Cartesian coordinate spaces, which are difficult for people to comprehend because of the high-dimensional space, an alternative to view data with four or more dimensions is using parallel coordinates. The parallel coordinates representation of a solution set can partly reflect data and can be an assistant tool (but not entirely replacing measurement indicators) in assessing a many-objective solution set. Figure 1 gives the solution sets obtained by six algorithms of five-objective MaF6. It can be clearly seen that MaOEA/DS has the best effect, followed by RVEA. The convergence and diversity of other algorithms are poor. MOEADD, SPEAR, and RVEA do not fully converge, while NSGAIII and onybyoneEA converge, but their diversity is relatively poor.

#### 3.3.2. WFG Suite

Table 4 reports the IGD mean and standard deviation values obtained by 6 MOEAs on 27 WFG test problems. It can be seen that MaOEA/DS showed obvious advantages on WFG3–7 and WFG9, while RVEA performed best on WFG1. Specifically, MaOEA/DS performs poorly for WFG1 with separable and unimodal problems, and NSGAIII performs best for WFG2 with a scaled disconnected Pareto front. For WFG3 with a degenerate Pareto front, MaOEA/DS performs best. In WFG4–9, they have larger “hill sizes”. In addition, WFG5 is a very deceptive problem, and the nonseparable reduction of WFG6 and WFG9 is more difficult. For WFG8, the distance-dependent parameters depend on the position-dependent parameters, which means that the optimizer cannot simply find a good set of distance parameters. Statistically, MaOEA/DS outperforms other algorithms on most test problems. Overall, the proposed MaOEA/DS algorithm outperforms NSGAIII, MOEADD, onebyoneEA, SPEAR, and RVEA on 17, 21, 24, 20, and 18 problems, respectively. These comparison results show that the dual selection strategy effectively balances the convergence and diversity.

Table 5 reports the PD mean and standard deviation values obtained by 6 MOEAs on 27 WFG test problems. Of the 27 problems, the statistical performance of MOEA/DS on 24 problems is better than that of the comparison algorithm. MOEA/DS performs better than NSGAIII, MOEA/DD, onebyoneEA, SPEAR, and RVEA on 24, 26, 25, 26, and 26 problems. Interestingly, NSGAIII performs best on WFG1, and for the remaining test problems, MaOEA/DS perform best, indicating that the proposed algorithm has good diversity, in which the PC strategy plays an important role.

**Figure 2:**
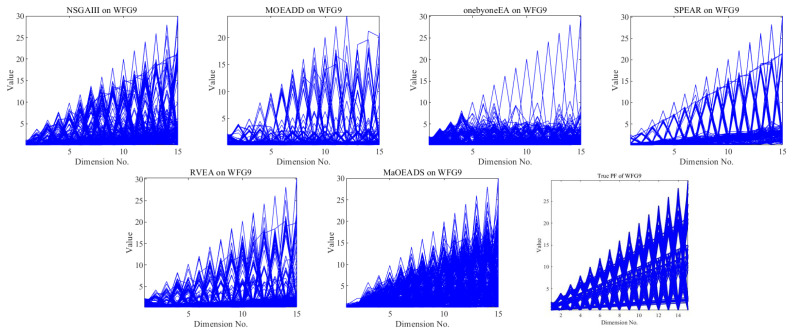
The final solution set obtained by the 6 MOEAs on 15-objective WFG9.

Figure 2 gives the solution sets obtained by 6 algorithms of 15-objective WFG9. It can be clearly seen that MaOEA/DS has the best effect and has been well diffused throughout the Pareto frontier, indicating its good convergence and diversity. Although other algorithms mostly converge, their diversity is poor, with the onebyoneEA algorithm having the worst diversity.

**Figure 3:**
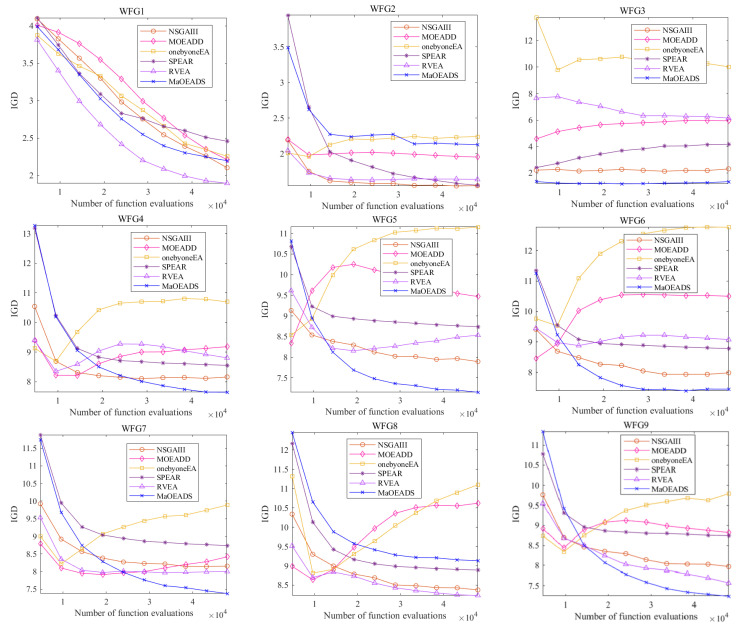
Evolution trajectory of IGD on all 15-objective WFG test problems (the error bars are very tiny due to low variance between replicates, so the figure does not include error bars).

Figure 3 shows the evolution trajectory of IGD on all 15-objective WFG test problems, where the horizontal coordinate represents the number of functional evaluations and the vertical coordinate represents the IGD value. This indicates the change trend of IGD value during evolution. As we can see, the proposed MaOEA/DS algorithm has obvious advantages in test problems except for WFG1, 2, and 8. In addition, there is an interesting phenomenon that after a certain number of functional evaluations, the evolutionary curves of MOEA/DD and onebyoneEA have an upward trend. For MOEA/DD, some solutions at the worst non-dominated level can be unconditionally retained for the next generation, changing the diversity of the population, so the IGD value has an upward trend. For onybyoneEA, in the early search stage, the focus is on convergence, while in the later search stage, the focus is on population diversity, resulting in reduced convergence, so the IGD value of onebyone increases.

### 3.4. Validation of Distance Function

In order to verify the advantages of the distance function proposed in this paper, it is compared with several distance-based algorithms: BiGE [40] (the Manhattan distance, L1-norm-based), KnEA (the Euclidean distance, L2-norm-based) [14], and Two_Arch2 [41] (Lp-norm-based (*p* < 1)). Table 6 reports the IGD mean and standard deviation values obtained by four MOEAs on 21 DTLZ test instances. By comparing the statistical results on 21 test instances, MaOEA/DS is superior to BiGE, KnEA, and Two_Arch2 on 18, 17, and 12 instances, respectively, and its performance is far better than BiGE and KnEA, but KnEA performs best on DTLZ7. The MaOEA/DS algorithm is slightly better than the Two_Arch2 algorithm, but the Two_Arch2 algorithm obtains the optimal result on the DTLZ1 problem. It can be seen that the Two_Arch2 algorithm still has advantages. Overall, MaOEA/DS performed best.

### 3.5. Analysis of the PC Strategy

The PC strategy proposed in this article is very important for environment selection, because it further selects the solution set with good diversity based on the diversity indictors. In order to verify the effectiveness of the PC strategy, we extend the PC strategy to NSGA-III to update the external population, and the resulting variant is expressed as NSGA-III-PC. Table 7 reports the average IGD values obtained during 20 independent runs on 5, 10, and 15 objective benchmarks. Of the 21 instances of the DTLZ test problems, NSGA-III-PC is superior to NSGA-III in 15 instances. It can be seen that the PC strategy is a general framework that can be integrated into other algorithms to improve the performance of the algorithm.

Here, we perform ablation studies on Equation (4) and do not use other strategies for verification. We only simply calculate the diversity of solutions to avoid the impact on the verification results. D1 denotes the first term of Equation (4) (i.e., $∥x^{i}-x^{j}{∥}_{2}$), and D2 denotes the whole equation. In Table 8, we report the average PD obtained by D1 and D2 in 20 independent runs on 5, 10, and 15 objective test benchmarks. The larger the PD value, the better the diversity. It can be seen that among the different objective numbers of DTLZ1–7 test problems, D2 is significantly better than D1 on 5, 7, and 5 test problems, respectively. It can be seen that the second term of Equation (4) is very important to improve the diversity of the solution set, because the second term also considers the difference between each component on the basis of the first term.

### 3.6. Analysis of Dual Selection

In Section 3.3, comparing the IGD values, it is evident that the proposed algorithm performs significantly better than other comparative algorithms, as in the first selection, the individual with the best convergence is selected from the top few individuals with good diversity and placed in the population, which narrows the range of optimal solutions. In the second selection, based on the PC strategy, further individuals are selected with better diversity. Comparing the PD values, it can be seen that the diversity of the proposed algorithm far exceeds that of other algorithms. This stems from the advantages of the PC strategy, as it not only considers the distance between the closest two points in the objective space, but also the differences between each objective function when evaluating the diversity of the solution set and considers the impact of the crowding degree of surrounding individuals. Compared to most other environmental selection strategies, the dual selection strategy does not require additional reference vectors and other parameter controls, and the obtained solution set is relatively stable.

## 4. Conclusions

Aiming at the existing problems of high-dimensional multi-objective evolution, this paper proposes a many-objective algorithm based on the dual selection strategy. First, a new distance function is designed as an effective distance metric. Then, a PC strategy is proposed to further enhance the algorithm’s ability to select superior solutions. Finally, a dual selection strategy is proposed. In the first selection, the individuals with the best convergence are selected from the top few individuals with good diversity in the population, focusing on population convergence. In the second selection, the PC strategy is used to further select individuals with larger crowding distance values, emphasizing population diversity. Extensive comparisons are made between MaOEA/DS and several state-of-the-art algorithms for 93 instances of 31 test problems from 3 well-known test suites on 5, 10, and 15 objectives. The results show that MaOEA/AS has better overall performance and is a promising MaOEA. In addition, the proposed PC strategy is combined with other advanced MaOPs methods. The results show that it is beneficial to improve the performance of other MaOEAs algorithms.

The work of this paper is obvious to improve the diversity, and the PC strategy can be extended to improve the diversity of other algorithms. In future work, the algorithm should be applied to different practical problems to further verify the effectiveness of the algorithm.

## Figures and Tables

**Table 1:** Parameter settings and test problem characteristics.

Problem	M	D	Characteristics
MaF1	5, 10, 15	M + K−1, K = 10	Linear, with an inverted Pareto front
MaF2	5, 10, 15	M + K−1, K = 10	Concave
MaF3	5, 10, 15	M + K−1, K = 10	Multi-modal, convex
MaF4	5, 10, 15	M + K−1, K = 10	Multi-modal, concave
MaF5	5, 10, 15	M + K−1, K = 10	Convex, biased
MaF6	5, 10, 15	M + K−1, K = 10	Concave, degenerate
MaF7	5, 10, 15	M + K−1, K = 20	Multi-model, mixed, disconnected
MaF8	5, 10, 15	2	Linear, degenerate
MaF9	5, 10, 15	2	Linear, degenerate
MaF10	5, 10, 15	M + K−1, K = 10	Mixed, biased
MaF11	5, 10, 15	M + K−1, K = 10	Convex, disconnected, non-separable
MaF12	5, 10, 15	M + K−1, K = 10	Concave, non-separable, biased, deceptive
MaF13	5, 10, 15	5	Concave, unimodal, non-separable, degenerate
MaF14	5, 10, 15	M * 20	Linear, partially separable, largescale
MaF15	5, 10, 15	M * 20	Convex, partially separable, largescale
DTLZ1	5, 10, 15	M + K−1, K = 5	Linear, multi-modal
DTLZ2	5, 10, 15	M + K−1, K = 10	Concave
DTLZ3	5, 10, 15	M + K−1, K = 10	Concave, multi-modal
DTLZ4	5, 10, 15	M + K−1, K = 10	Concave, biased
DTLZ5	5, 10, 15	M + K−1, K = 10	Concave, degenerate
DTLZ6	5, 10, 15	M + K−1, K = 10	Concave, degenerate, biased
DTLZ7	5, 10, 15	M + K−1, k = 20	Mixed, disconnected, multi-modal, scaled
WFG1	5, 10, 15	M + L − 1, L = 10	Mixed, biased, scaled
WFG2	5, 10, 15	M + L − 1, L = 10	Convex, disconnected, multi-modal,
			non-separable, scaled
WFG3	5, 10, 15	M + L − 1, L = 10	Linear, degenerate, non-separable, scaled
WFG4	5, 10, 15	M + L − 1, L = 10	Concave, multi-modal, scaled
WFG5	5, 10, 15	M + L − 1, L = 10	Concave, biased, scaled
WFG6	5, 10, 15	M + L − 1, L = 10	Concave, non-separable, scaled
WFG7	5, 10, 15	M + L − 1, L = 10	Concave, biased, scaled
WFG8	5, 10, 15	M + L − 1, L = 10	Concave, biased, non-separable, scaled
WFG9	5, 10, 15	M + L − 1, L = 10	Concave, biased, multi-modal,
			deceptive, non-separable, scaled

**Table 2:** Comparison of IGD values of six algorithms on MaF test problems.

Problems	M	NSGAIII	MOEA/DD	1by1EA	SPEAR	RVEA	MaOEADS
MaF1	5	$1.8345\times 10^{-1}$ ($1.02\times 10^{-2}$)−	$2.0954\times 10^{-1}$ ($3.46\times 10^{-3}$)−	$1.0163\times 10^{-1}$ ($1.57\times 10^{-3}$)−	$1.8912\times 10^{-1}$ ($1.33\times 10^{-3}$)−	$2.7142\times 10^{-1}$ ($1.49\times 10^{-2}$)−	$9.1426\times 10^{-2}$ ($7.17\times 10^{-4}$)
	10	$2.7851\times 10^{-1}$ ($2.98\times 10^{-3}$)−	$4.7343\times 10^{-1}$ ($1.88\times 10^{-2}$)−	$3.2940\times 10^{-1}$ ($6.83\times 10^{-2}$)−	$4.5138\times 10^{-1}$ ($3.80\times 10^{-2}$)−	$5.8318\times 10^{-1}$ ($5.44\times 10^{-2}$)−	$1.8858\times 10^{-1}$ ($1.63\times 10^{-3}$)
	15	$3.1709\times 10^{-1}$ ($4.91\times 10^{-3}$)−	$5.4376\times 10^{-1}$ ($2.95\times 10^{-2}$)−	$4.6163\times 10^{-1}$ ($3.50\times 10^{-2}$)−	$6.7984\times 10^{-1}$ ($1.02\times 10^{-1}$)−	$6.9034\times 10^{-1}$ ($4.94\times 10^{-2}$)−	$2.3064\times 10^{-1}$ ($1.63\times 10^{-3}$)
MaF2	5	$1.1146\times 10^{-1}$ ($2.52\times 10^{-3}$)−	$1.2967\times 10^{-1}$ ($3.39\times 10^{-3}$)−	$8.1707\times 10^{-2}$ ($1.64\times 10^{-3}$)−	$1.1870\times 10^{-1}$ ($9.00\times 10^{-4}$)−	$1.1577\times 10^{-1}$ ($1.09\times 10^{-3}$)−	$7.7596\times 10^{-2}$ ($1.97\times 10^{-3}$)
	10	$2.0163\times 10^{-1}$ ($1.58\times 10^{-2}$)−	$2.5272\times 10^{-1}$ ($3.17\times 10^{-2}$)−	$2.6154\times 10^{-1}$ ($2.24\times 10^{-2}$)−	$2.0604\times 10^{-1}$ ($5.08\times 10^{-3}$)−	$2.5083\times 10^{-1}$ ($3.43\times 10^{-2}$)−	$1.4983\times 10^{-1}$ ($1.53\times 10^{-3}$)
	15	$2.0525\times 10^{-1}$ ($6.45\times 10^{-3}$)−	$4.1640\times 10^{-1}$ ($3.63\times 10^{-2}$)−	$4.8877\times 10^{-1}$ ($3.43\times 10^{-2}$)−	$4.4203\times 10^{-1}$ ($6.55\times 10^{-2}$)−	$5.1266\times 10^{-1}$ ($1.69\times 10^{-1}$)−	$1.7458\times 10^{-1}$ ($1.52\times 10^{-3}$)
MaF3	5	$7.5927\times 10^{-2}$ ($6.74\times 10^{-3}$) +	$9.8271\times 10^{-2}$ ($5.33\times 10^{-3}$)+	$1.5959\times 10^{-1}$ ($2.33\times 10^{-2}$)+	$9.8271\times 10^{0}$ ($1.13\times 10^{1}$)−	$2.5097\times 10^{-1}$ ($6.07\times 10^{-1}$)+	$4.5595\times 10^{0}$ ($5.72\times 10^{0}$)
	10	$1.5189\times 10^{3}$ ($1.64\times 10^{3}$)−	$5.4546\times 10^{-1}$ ($1.67\times 10^{0}$)+	$1.6063\times 10^{-1}$ ($1.39\times 10^{-1}$)+	$7.3295\times 10^{5}$ ($2.37\times 10^{6}$)−	$1.1948\times 10^{-1}$ ($1.32\times 10^{-2}$)+	$1.3143\times 10^{0}$ ($1.38\times 10^{0}$)
	15	$3.7160\times 10^{2}$ ($7.74\times 10^{2}$)−	$6.0721\times 10^{-1}$ ($9.22\times 10^{-1}$)=	$9.8368\times 10^{-1}$ ($1.77\times 10^{0}$)=	$5.9275\times 10^{6}$ ($1.46\times 10^{7}$)−	$1.2125\times 10^{-1}$ ($9.50\times 10^{-2}$)+	$5.6069\times 10^{-1}$ ($9.85\times 10^{-1}$)
MaF4	5	$2.4726\times 10^{0}$ ($5.59\times 10^{-1}$)−	$5.5061\times 10^{0}$ ($5.96\times 10^{-1}$)−	$5.6277\times 10^{0}$ ($8.77\times 10^{-1}$)−	$5.5966\times 10^{0}$ ($2.05\times 10^{0}$)−	$3.3995\times 10^{0}$ ($4.66\times 10^{-1}$)−	$1.7721\times 10^{0}$ ($5.19\times 10^{-2}$)
	10	$9.2926\times 10^{1}$ ($7.51\times 10^{0}$)−	$3.9287\times 10^{2}$ ($1.14\times 10^{1}$)−	$2.4858\times 10^{2}$ ($4.70\times 10^{1}$)−	$5.4101\times 10^{2}$ ($5.82\times 10^{2}$)−	$2.0887\times 10^{2}$ ($5.55\times 10^{1}$)−	$5.5409\times 10^{1}$ ($5.18\times 10^{0}$)
	15	$3.8655\times 10^{3}$ ($2.56\times 10^{2}$)−	$1.4061\times 10^{+4}$ ($2.89\times 10^{3}$)−	$1.0856\times 10^{+4}$ ($4.31\times 10^{2}$)−	$1.0386\times 10^{+5}$ ($6.85\times 10^{+4}$)−	$8.5214\times 10^{3}$ ($1.99\times 10^{3}$)−	$2.1546\times 10^{3}$ ($1.19\times 10^{3}$)
MaF5	5	$1.9699\times 10^{0}$ ($3.12\times 10^{-3}$)+	$3.9913\times 10^{0}$ ($4.41\times 10^{-1}$)+	$3.9276\times 10^{0}$ ($6.95\times 10^{-1}$)+	$1.9995\times 10^{0}$ ($9.40\times 10^{-3}$)+	$1.9803\times 10^{0}$ ($5.49\times 10^{-2}$)+	$9.4326\times 10^{0}$ ($2.49\times 10^{0}$)
	10	$7.7422\times 10^{1}$ ($1.01\times 10^{0}$)−	$2.9133\times 10^{2}$ ($1.53\times 10^{1}$)−	$1.9743\times 10^{2}$ ($1.57\times 10^{1}$)−	$8.0777\times 10^{1}$ ($3.44\times 10^{0}$)−	$9.2979\times 10^{1}$ ($9.82\times 10^{0}$)−	$5.4744\times 10^{1}$ ($2.46\times 10^{1}$)
	15	$2.4609\times 10^{3}$ ($9.90\times 10^{1}$)+	$7.2550\times 10^{3}$ ($1.92\times 10^{2}$)−	$6.0073\times 10^{3}$ ($6.09\times 10^{1}$)−	$2.3936\times 10^{3}$ ($3.04\times 10^{2}$) +	$3.1436\times 10^{3}$ ($4.70\times 10^{2}$)=	$2.8808\times 10^{3}$ ($5.28\times 10^{2}$)
MaF6	5	$1.6723\times 10^{-2}$ ($3.28\times 10^{-3}$)−	$6.8523\times 10^{-2}$ ($4.22\times 10^{-3}$)−	$2.1072\times 10^{-3}$ ($3.91\times 10^{-5}$)−	$8.7946\times 10^{-2}$ ($1.06\times 10^{-2}$)−	$7.2886\times 10^{-2}$ ($1.07\times 10^{-2}$)−	$1.3447\times 10^{-3}$ ($1.06\times 10^{-5}$)
	10	$6.5840\times 10^{-1}$ ($3.15\times 10^{-1}$)−	$9.6544\times 10^{-2}$ ($1.41\times 10^{-2}$)−	$1.5991\times 10^{-3}$ ($2.03\times 10^{-5}$)−	$2.0470\times 10^{-1}$ ($5.51\times 10^{-2}$)−	$1.2223\times 10^{-1}$ ($1.66\times 10^{-2}$)−	$1.0249\times 10^{-3}$ ($7.98\times 10^{-6}$)
	15	$8.1306\times 10^{-1}$ ($4.19\times 10^{-1}$)−	$1.2535\times 10^{-1}$ ($5.66\times 10^{-3}$)−	$1.8367\times 10^{-3}$ ($2.17\times 10^{-5}$) +	$1.4864\times 10^{1}$ ($2.67\times 10^{1}$)-	$3.1968\times 10^{-1}$ ($2.64\times 10^{-1}$)−	$8.7607\times 10^{-3}$ ($3.03\times 10^{-2}$)
MaF7	5	$2.8042\times 10^{-1}$ ($8.41\times 10^{-3}$)+	$2.7707\times 10^{0}$ ($7.04\times 10^{-1}$)−	$3.1908\times 10^{-1}$ ($2.93\times 10^{-2}$)+	$3.5624\times 10^{-1}$ ($4.61\times 10^{-3}$)+	$5.0036\times 10^{-1}$ ($8.94\times 10^{-3}$)+	$1.4840\times 10^{0}$ ($5.80\times 10^{-3}$)
	10	$1.0373\times 10^{0}$ ($6.70\times 10^{-2}$)+	$2.6091\times 10^{0}$ ($3.24\times 10^{-1}$)+	$2.2874\times 10^{0}$ ($6.08\times 10^{-1}$)+	$2.0232\times 10^{0}$ ($1.41\times 10^{-2}$)+	$2.1306\times 10^{0}$ ($4.08\times 10^{-1}$)+	$3.2525\times 10^{0}$ ($4.91\times 10^{-2}$)
	15	$4.4230\times 10^{0}$ ($5.33\times 10^{-1}$)+	$3.4683\times 10^{0}$ ($3.70\times 10^{-2}$)+	$3.3704\times 10^{0}$ ($4.89\times 10^{-1}$)+	$1.6740\times 10^{1}$ ($1.07\times 10^{1}$)−	$3.0298\times 10^{0}$ ($4.32\times 10^{-1}$)+	$7.2025\times 10^{0}$ ($2.28\times 10^{-1}$)
MaF8	5	$1.5775\times 10^{-1}$ ($9.30\times 10^{-3}$)=	$2.7897\times 10^{-1}$ ($1.97\times 10^{-2}$)−	$3.5483\times 10^{-1}$ ($6.98\times 10^{-2}$)−	$5.3870\times 10^{2}$ ($9.37\times 10^{2}$)−	$3.0995\times 10^{-1}$ ($2.81\times 10^{-2}$)−	$2.1188\times 10^{-1}$ ($1.39\times 10^{-1}$)
	10	$3.6039\times 10^{-1}$ ($7.04\times 10^{-2}$)−	$9.1389\times 10^{-1}$ ($1.53\times 10^{-2}$)−	$3.3020\times 10^{-1}$ ($4.78\times 10^{-2}$)−	$4.1511\times 10^{2}$ ($7.78\times 10^{2}$)−	$9.5080\times 10^{-1}$ ($1.13\times 10^{-1}$)−	$1.9272\times 10^{-1}$ ($1.49\times 10^{-1}$)
	15	$4.0248\times 10^{-1}$ ($8.14\times 10^{-2}$)−	$1.3230\times 10^{0}$ ($4.24\times 10^{-2}$)−	$3.8803\times 10^{-1}$ ($6.64\times 10^{-2}$)−	$5.2170\times 10^{2}$ ($8.75\times 10^{2}$)−	$1.3186\times 10^{0}$ ($2.41\times 10^{-1}$)−	$3.3648\times 10^{-1}$ ($2.86\times 10^{-1}$)
MaF9	5	$3.7274\times 10^{-1}$ ($1.72\times 10^{-1}$)−	$2.2488\times 10^{-1}$ ($2.96\times 10^{-3}$)−	$1.4953\times 10^{-1}$ ($3.88\times 10^{-2}$)−	$9.0486\times 10^{-1}$ ($2.55\times 10^{-1}$)−	$2.8671\times 10^{-1}$ ($4.34\times 10^{-2}$)−	$1.2036\times 10^{-1}$ ($5.62\times 10^{-2}$)
	10	$4.9636\times 10^{-1}$ ($8.99\times 10^{-2}$)−	$5.9556\times 10^{-1}$ ($1.37\times 10^{-3}$)−	$1.1363\times 10^{-1}$ ($6.91\times 10^{-3}$)−	$3.3454\times 10^{0}$ ($5.02\times 10^{0}$)−	$9.5706\times 10^{-1}$ ($1.80\times 10^{-1}$)−	$9.4893\times 10^{-2}$ ($3.09\times 10^{-3}$)
	15	$3.8037\times 10^{-1}$ ($5.77\times 10^{-2}$)−	$9.5971\times 10^{-1}$ ($8.40\times 10^{-3}$)−	$1.8964\times 10^{-1}$ ($1.08\times 10^{-1}$)−	$9.4826\times 10^{0}$ ($8.93\times 10^{0}$)−	$1.3899\times 10^{0}$ ($2.41\times 10^{-1}$)−	$1.5935\times 10^{-1}$ ($6.06\times 10^{-2}$)
MaF10	5	$3.6920\times 10^{-1}$ ($5.36\times 10^{-3}$)+	$4.6014\times 10^{-1}$ ($2.80\times 10^{-2}$)+	$7.1500\times 10^{-1}$ ($3.80\times 10^{-2}$)−	$3.7513\times 10^{-1}$ ($9.08\times 10^{-3}$)+	$3.7971\times 10^{-1}$ ($1.62\times 10^{-2}$)+	$4.7631\times 10^{-1}$ ($2.21\times 10^{-2}$)
	10	$1.0328\times 10^{0}$ ($6.37\times 10^{-2}$)+	$1.3668\times 10^{0}$ ($4.03\times 10^{-2}$)=	$1.7936\times 10^{0}$ ($3.41\times 10^{-2}$)−	$1.3269\times 10^{0}$ ($9.56\times 10^{-2}$)=	$1.1056\times 10^{0}$ ($4.06\times 10^{-2}$)+	$1.3562\times 10^{0}$ ($1.27\times 10^{-1}$)
	15	$1.5889\times 10^{0}$ ($8.03\times 10^{-2}$)+	$1.9856\times 10^{0}$ ($3.63\times 10^{-2}$)−	$2.4284\times 10^{0}$ ($4.36\times 10^{-2}$)−	$1.8819\times 10^{0}$ ($9.29\times 10^{-2}$)=	$1.6686\times 10^{0}$ ($4.28\times 10^{-2}$)+	$1.9067\times 10^{0}$ ($8.31\times 10^{-2}$)
MaF11	5	$3.8913\times 10^{-1}$ ($1.80\times 10^{-3}$)+	$5.1030\times 10^{-1}$ ($1.26\times 10^{-2}$)−	$6.6779\times 10^{-1}$ ($5.95\times 10^{-2}$)−	$3.9485\times 10^{-1}$ ($2.96\times 10^{-3}$)+	$3.8303\times 10^{-1}$ ($8.59\times 10^{-3}$)+	$4.3126\times 10^{-1}$ ($4.60\times 10^{-2}$)
	10	$1.2442\times 10^{0}$ ($1.32\times 10^{-1}$)=	$1.4645\times 10^{0}$ ($2.26\times 10^{-2}$)−	$1.8264\times 10^{0}$ ($6.85\times 10^{-2}$)−	$1.0825\times 10^{0}$ ($1.01\times 10^{-2}$)+	$1.0938\times 10^{0}$ ($3.20\times 10^{-2}$)+	$1.2951\times 10^{0}$ ($5.00\times 10^{-2}$)
	15	$1.5552\times 10^{0}$ ($7.22\times 10^{-2}$)+	$1.9170\times 10^{0}$ ($4.71\times 10^{-2}$)=	$2.3724\times 10^{0}$ ($8.88\times 10^{-2}$)−	$1.4537\times 10^{0}$ ($1.73\times 10^{-2}$)+	$1.7625\times 10^{0}$ ($1.18\times 10^{-1}$)+	$1.9982\times 10^{0}$ ($1.66\times 10^{-1}$)
MaF12	5	$9.3413\times 10^{-1}$ ($3.32\times 10^{-3}$)−	$1.0323\times 10^{0}$ ($4.53\times 10^{-3}$)−	$1.4009\times 10^{0}$ ($1.07\times 10^{-1}$)−	$9.4416\times 10^{-1}$ ($3.13\times 10^{-3}$)−	$9.4320\times 10^{-1}$ ($1.25\times 10^{-3}$)−	$8.3780\times 10^{-1}$ ($6.30\times 10^{-3}$)
	10	$4.4110\times 10^{0}$ ($3.55\times 10^{-2}$)−	$6.2937\times 10^{0}$ ($2.33\times 10^{-1}$)−	$5.4833\times 10^{0}$ ($2.50\times 10^{-1}$)−	$4.5182\times 10^{0}$ ($1.52\times 10^{-2}$)−	$4.2530\times 10^{0}$ ($4.64\times 10^{-2}$)−	$3.6907\times 10^{0}$ ($3.57\times 10^{-2}$)
	15	$7.9322\times 10^{0}$ ($1.55\times 10^{-1}$)−	$8.6002\times 10^{0}$ ($9.73\times 10^{-2}$)−	$9.8737\times 10^{0}$ ($2.52\times 10^{-1}$)−	$8.4960\times 10^{0}$ ($1.27\times 10^{-1}$)−	$7.4625\times 10^{0}$ ($2.72\times 10^{-1}$)−	$6.9647\times 10^{0}$ ($1.76\times 10^{-1}$)
MaF13	5	$2.0432\times 10^{-1}$ ($2.25\times 10^{-2}$)−	$2.0898\times 10^{-1}$ ($5.52\times 10^{-2}$)−	$8.2507\times 10^{-2}$ ($4.60\times 10^{-3}$)+	$4.1492\times 10^{-1}$ ($1.18\times 10^{-1}$)−	$3.7273\times 10^{-1}$ ($5.15\times 10^{-2}$)−	$1.0091\times 10^{-1}$ ($1.12\times 10^{-2}$)
	10	$2.4480\times 10^{-1}$ ($1.53\times 10^{-2}$)−	$3.5242\times 10^{-1}$ ($2.84\times 10^{-2}$)−	$1.2224\times 10^{-1}$ ($9.64\times 10^{-3}$)=	$5.9085\times 10^{-1}$ ($2.24\times 10^{-1}$)−	$7.4754\times 10^{-1}$ ($2.33\times 10^{-1}$)−	$1.2418\times 10^{-1}$ ($1.16\times 10^{-2}$)
	15	$2.9572\times 10^{-1}$ ($5.52\times 10^{-2}$)−	$3.9552\times 10^{-1}$ ($3.51\times 10^{-2}$)−	$2.3828\times 10^{-1}$ ($5.18\times 10^{-2}$)−	$8.2636\times 10^{-1}$ ($3.09\times 10^{-1}$)−	$9.9778\times 10^{-1}$ ($3.30\times 10^{-1}$)−	$1.5684\times 10^{-1}$ ($1.62\times 10^{-2}$)
MaF14	5	$1.6886\times 10^{0}$ ($9.20\times 10^{-1}$)−	$7.1960\times 10^{-1}$ ($1.31\times 10^{-1}$)=	$4.6061\times 10^{-1}$ ($8.63\times 10^{-2}$)+	$1.5536\times 10^{0}$ ($4.03\times 10^{-1}$)−	$1.0467\times 10^{0}$ ($3.51\times 10^{-1}$)−	$8.0980\times 10^{-1}$ ($3.89\times 10^{-1}$)
	10	$1.0122\times 10^{1}$ ($3.72\times 10^{0}$)−	$1.1579\times 10^{0}$ ($1.10\times 10^{-1}$)+	$1.1651\times 10^{0}$ ($1.83\times 10^{-1}$)+	$1.4573\times 10^{1}$ ($3.64\times 10^{0}$)−	$1.0298\times 10^{0}$ ($6.80\times 10^{-2}$)+	$2.4867\times 10^{0}$ ($2.59\times 10^{0}$)
	15	$3.1051\times 10^{0}$ ($2.52\times 10^{0}$)−	$1.4145\times 10^{0}$ ($3.11\times 10^{-1}$)=	$2.9030\times 10^{0}$ ($1.01\times 10^{0}$)−	$1.4737\times 10^{1}$ ($8.27\times 10^{0}$)−	$2.3754\times 10^{0}$ ($1.51\times 10^{0}$)−	$1.6806\times 10^{0}$ ($1.02\times 10^{0}$)
MaF15	5	$1.1786\times 10^{0}$ ($1.79\times 10^{-1}$)−	$4.8122\times 10^{-1}$ ($2.43\times 10^{-2}$)+	$6.0091\times 10^{-1}$ ($5.94\times 10^{-2}$)+	$1.3722\times 10^{0}$ ($5.21\times 10^{-1}$)−	$5.5401\times 10^{-1}$ ($4.63\times 10^{-2}$)+	$7.4909\times 10^{-1}$ ($6.19\times 10^{-2}$)
	10	$2.5515\times 10^{0}$ ($1.46\times 10^{0}$)−	$1.0883\times 10^{0}$ ($7.25\times 10^{-2}$)=	$1.0845\times 10^{0}$ ($7.79\times 10^{-2}$)=	$1.2694\times 10^{1}$ ($4.34\times 10^{0}$)−	$1.0466\times 10^{0}$ ($5.05\times 10^{-2}$)+	$1.0886\times 10^{0}$ ($5.30\times 10^{-2}$)
	15	$1.1451\times 10^{1}$ ($3.81\times 10^{0}$)−	$1.5393\times 10^{0}$ ($1.89\times 10^{-1}$)-	$1.5073\times 10^{0}$ ($8.67\times 10^{-2}$)−	$3.9782\times 10^{1}$ ($7.27\times 10^{0}$)−	$1.2747\times 10^{0}$ ($4.96\times 10^{-2}$)=	$1.2560\times 10^{0}$ ($4.49\times 10^{-2}$)
+/−/=		11/32/2	8/31/6	11/31/3	8/35/2	16/27/2	-−−−−−

Note: Bold marks indicate the best-performing results.

**Table 3:** Comparison of PD values of five algorithms on MaF test problems.

Problems	M	NSGAIII	MOEA/DD	1by1EA	RVEA	MaOEADS
MaF1	5	$3.3319\times 10^{7}$ ($1.81\times 10^{6}$)−	$1.1208\times 10^{6}$ ($4.79\times 10^{5}$)−	$5.3863\times 10^{7}$ ($1.14\times 10^{6}$)−	$6.6932\times 10^{6}$ ($2.72\times 10^{6}$)−	$7.0322\times 10^{7}$ ($2.01\times 10^{6}$)
	10	$2.3081\times 10^{10}$ ($1.78\times 10^{9}$)−	$5.7513\times 10^{8}$ ($5.49\times 10^{8}$)−	$2.2527\times 10^{10}$ ($1.22\times 10^{10}$)−	$1.1589\times 10^{9}$ ($7.97\times 10^{8}$)−	$6.4133\times 10^{10}$ ($3.73\times 10^{9}$)
	15	$1.3270\times 10^{12}$ ($1.09\times 10^{11}$)−	$7.9596\times 10^{9}$ ($6.21\times 10^{9}$)−	$2.1384\times 10^{11}$ ($6.55\times 10^{10}$)−	$5.7370\times 10^{10}$ ($4.06\times 10^{10}$)−	$2.0571\times 10^{12}$ ($8.89\times 10^{10}$)
MaF2	5	$3.2270\times 10^{7}$ ($1.67\times 10^{6}$)−	$1.6717\times 10^{7}$ ($1.33\times 10^{6}$)−	$4.4457\times 10^{7}$ ($1.36\times 10^{6}$)−	$2.4069\times 10^{7}$ ($9.99\times 10^{5}$)−	$6.5890\times 10^{7}$ ($1.41\times 10^{6}$)
	10	$3.0515\times 10^{10}$ ($1.84\times 10^{9}$)−	$1.0329\times 10^{10}$ ($1.57\times 10^{9}$)−	$5.0090\times 10^{10}$ ($2.70\times 10^{9}$)−	$1.0180\times 10^{10}$ ($9.06\times 10^{8}$)−	$8.3065\times 10^{10}$ ($1.38\times 10^{9}$)
	15	$1.1881\times 10^{12}$ ($6.55\times 10^{10}$)−	$1.0085\times 10^{11}$ ($1.63\times 10^{10}$)−	$9.1750\times 10^{11}$ ($1.77\times 10^{11}$)−	$3.6893\times 10^{11}$ ($1.25\times 10^{11}$)−	$2.9968\times 10^{12}$ ($4.74\times 10^{10}$)
MaF3	5	$5.8257\times 10^{8}$ ($1.97\times 10^{9}$)−	$1.3061\times 10^{7}$ ($7.91\times 10^{5}$)−	$1.1743\times 10^{9}$ ($2.88\times 10^{9}$)−	$5.2266\times 10^{12}$ ($1.71\times 10^{13}$)−	$2.5026\times 10^{16}$ ($4.97\times 10^{16}$)
	10	$2.1425\times 10^{16}$ ($5.07\times 10^{16}$)=	$3.2286\times 10^{14}$ ($7.66\times 10^{14}$)−	$2.3644\times 10^{15}$ ($9.37\times 10^{15}$)−	$1.8866\times 10^{14}$ ($6.39\times 10^{14}$)−	$3.0055\times 10^{17}$ ($5.99\times 10^{17}$)
	15	$2.7534\times 10^{19}$ ($1.58\times 10^{19}$)+	$1.1964\times 10^{18}$ ($4.22\times 10^{18}$)=	$6.9661\times 10^{16}$ ($2.27\times 10^{17}$)=	$3.5430\times 10^{17}$ ($4.72\times 10^{17}$)=	$9.1643\times 10^{18}$ ($3.51\times 10^{19}$)
MaF4	5	$7.7320\times 10^{8}$ ($8.45\times 10^{8}$)−	$2.3094\times 10^{8}$ ($7.00\times 10^{8}$)−	$3.9203\times 10^{8}$ ($3.38\times 10^{7}$)−	$5.5679\times 10^{9}$ ($4.88\times 10^{9}$)+	$8.4868\times 10^{8}$ ($4.02\times 10^{7}$)
	10	$1.5856\times 10^{12}$ ($1.44\times 10^{11}$)−	$5.2602\times 10^{10}$ ($2.42\times 10^{10}$)−	$2.1898\times 10^{12}$ ($4.96\times 10^{12}$)−	$6.4846\times 10^{10}$ ($4.16\times 10^{10}$)−	$7.3816\times 10^{12}$ ($8.67\times 10^{11}$)
	15	$4.1146\times 10^{14}$ ($5.20\times 10^{13}$)−	$6.0924\times 10^{12}$ ($3.75\times 10^{12}$)−	$1.3627\times 10^{14}$ ($4.67\times 10^{13}$)−	$4.4447\times 10^{15}$ ($5.56\times 10^{15}$)=	$2.5553\times 10^{15}$ ($3.58\times 10^{14}$)
MaF5	5	$4.2557\times 10^{7}$ ($2.61\times 10^{6}$)+	$3.4479\times 10^{7}$ ($1.33\times 10^{7}$)+	$1.8283\times 10^{8}$ ($1.77\times 10^{7}$)+	$5.9223\times 10^{7}$ ($9.46\times 10^{6}$)+	$3.9550\times 10^{5}$ ($3.60\times 10^{5}$)
	10	$7.1406\times 10^{11}$ ($2.57\times 10^{10}$)−	$4.4412\times 10^{10}$ ($7.61\times 10^{9}$)−	$1.3170\times 10^{11}$ ($2.53\times 10^{10}$)−	$2.6867\times 10^{10}$ ($1.86\times 10^{10}$)−	$1.7981\times 10^{12}$ ($6.61\times 10^{11}$)
	15	$4.5741\times 10^{14}$ ($2.87\times 10^{13}$)+	$1.0275\times 10^{12}$ ($4.30\times 10^{11}$)−	$3.1100\times 10^{12}$ ($9.00\times 10^{11}$)−	$5.1502\times 10^{12}$ ($1.91\times 10^{12}$)−	$5.3457\times 10^{13}$ ($2.84\times 10^{13}$)
MaF6	5	$7.6536\times 10^{6}$ ($8.79\times 10^{5}$)−	$2.2629\times 10^{9}$ ($5.13\times 10^{8}$)+	$8.9244\times 10^{6}$ ($1.22\times 10^{6}$)−	$1.0098\times 10^{9}$ ($6.10\times 10^{8}$)+	$9.8181\times 10^{6}$ ($1.06\times 10^{6}$)
	10	$2.7420\times 10^{11}$ ($1.31\times 10^{11}$)+	$2.4333\times 10^{11}$ ($1.50\times 10^{11}$)+	$3.9959\times 10^{9}$ ($5.03\times 10^{8}$)=	$3.6042\times 10^{10}$ ($3.59\times 10^{10}$)=	$4.3752\times 10^{9}$ ($5.05\times 10^{8}$)
	15	$2.6140\times 10^{13}$ ($5.10\times 10^{12}$)+	$1.8108\times 10^{13}$ ($7.54\times 10^{12}$)+	$1.0301\times 10^{11}$ ($1.74\times 10^{10}$)=	$6.1231\times 10^{12}$ ($8.98\times 10^{12}$)=	$1.0215\times 10^{12}$ ($3.65\times 10^{12}$)
MaF7	5	$3.3163\times 10^{7}$ ($4.01\times 10^{6}$)+	$6.9946\times 10^{5}$ ($2.25\times 10^{6}$)−	$5.0527\times 10^{7}$ ($2.72\times 10^{6}$)+	$2.0499\times 10^{7}$ ($2.26\times 10^{6}$)−	$2.6163\times 10^{7}$ ($1.30\times 10^{6}$)
	10	$3.0412\times 10^{10}$ ($2.98\times 10^{9}$)=	$2.5230\times 10^{9}$ ($1.50\times 10^{9}$)−	$1.9470\times 10^{10}$ ($3.82\times 10^{9}$)−	$1.4350\times 10^{10}$ ($1.66\times 10^{9}$)−	$3.2261\times 10^{10}$ ($3.57\times 10^{9}$)
	15	$8.2819\times 10^{11}$ ($9.20\times 10^{10}$)−	$2.7418\times 10^{10}$ ($7.61\times 10^{9}$)−	$8.6616\times 10^{11}$ ($2.20\times 10^{11}$)−	$5.5074\times 10^{11}$ ($6.71\times 10^{10}$)−	$1.7344\times 10^{12}$ ($1.09\times 10^{11}$)
MaF8	5	$5.5114\times 10^{7}$ ($2.87\times 10^{6}$)−	$2.7926\times 10^{7}$ ($3.74\times 10^{6}$)−	$4.5399\times 10^{7}$ ($7.01\times 10^{6}$)−	$4.5154\times 10^{7}$ ($4.82\times 10^{7}$)−	$7.6084\times 10^{7}$ ($2.13\times 10^{7}$)
	10	$5.8416\times 10^{10}$ ($6.24\times 10^{9}$)−	$8.4146\times 10^{9}$ ($1.83\times 10^{9}$)−	$7.9898\times 10^{10}$ ($4.72\times 10^{9}$)−	$3.0145\times 10^{9}$ ($4.42\times 10^{9}$)−	$1.0400\times 10^{11}$ ($3.53\times 10^{10}$)
	15	$3.4310\times 10^{12}$ ($3.87\times 10^{11}$)−	$3.7424\times 10^{11}$ ($7.91\times 10^{10}$)−	$4.7257\times 10^{12}$ ($3.34\times 10^{11}$)−	$1.6260\times 10^{15}$ ($1.34\times 10^{15}$)+	$6.1421\times 10^{12}$ ($1.22\times 10^{12}$)
MaF9	5	$1.1741\times 10^{9}$ ($1.51\times 10^{9}$)=	$2.4433\times 10^{9}$ ($3.23\times 10^{9}$)+	$1.4494\times 10^{8}$ ($2.83\times 10^{8}$)−	$9.2137\times 10^{8}$ ($1.49\times 10^{9}$)−	$1.3049\times 10^{9}$ ($1.41\times 10^{9}$)
	10	$4.2174\times 10^{13}$ ($5.62\times 10^{12}$)+	$1.9709\times 10^{11}$ ($7.54\times 10^{11}$)−	$5.1355\times 10^{11}$ ($6.33\times 10^{11}$)−	$2.1073\times 10^{11}$ ($7.12\times 10^{11}$)−	$2.1683\times 10^{12}$ ($1.56\times 10^{12}$)
	15	$7.2697\times 10^{14}$ ($4.58\times 10^{14}$)=	$5.7323\times 10^{13}$ ($2.20\times 10^{13}$)−	$3.1115\times 10^{13}$ ($5.54\times 10^{13}$)−	$9.4348\times 10^{13}$ ($1.34\times 10^{14}$)−	$5.0343\times 10^{14}$ ($2.79\times 10^{14}$)
MaF10	5	$6.4730\times 10^{7}$ ($7.85\times 10^{6}$)−	$7.6450\times 10^{7}$ ($5.13\times 10^{6}$)−	$6.7780\times 10^{7}$ ($3.08\times 10^{6}$)−	$7.0786\times 10^{7}$ ($9.16\times 10^{6}$)−	$1.1332\times 10^{8}$ ($8.62\times 10^{6}$)
	10	$8.3836\times 10^{10}$ ($1.04\times 10^{10}$)−	$3.9090\times 10^{10}$ ($4.13\times 10^{9}$)−	$4.2543\times 10^{10}$ ($4.45\times 10^{9}$)−	$4.4973\times 10^{10}$ ($4.28\times 10^{9}$)−	$1.2945\times 10^{11}$ ($2.05\times 10^{10}$)
	15	$1.9522\times 10^{12}$ ($2.34\times 10^{11}$)−	$5.9587\times 10^{11}$ ($1.06\times 10^{11}$)−	$1.7360\times 10^{12}$ ($9.32\times 10^{10}$)−	$1.1251\times 10^{12}$ ($1.15\times 10^{11}$)−	$3.7665\times 10^{12}$ ($2.71\times 10^{11}$)
MaF11	5	$7.8833\times 10^{7}$ ($3.18\times 10^{6}$)−	$7.3497\times 10^{7}$ ($2.02\times 10^{6}$)−	$8.5879\times 10^{7}$ ($3.75\times 10^{6}$)−	$9.2926\times 10^{7}$ ($3.22\times 10^{6}$)−	$1.6196\times 10^{8}$ ($6.92\times 10^{6}$)
	10	$6.2638\times 10^{10}$ ($1.22\times 10^{10}$)−	$3.9313\times 10^{10}$ ($1.98\times 10^{9}$)−	$6.7873\times 10^{10}$ ($1.86\times 10^{9}$)−	$5.2430\times 10^{10}$ ($3.16\times 10^{9}$)−	$1.4095\times 10^{11}$ ($5.54\times 10^{9}$)
	15	$3.3836\times 10^{12}$ ($4.55\times 10^{11}$)−	$6.4481\times 10^{11}$ ($7.02\times 10^{10}$)−	$2.4917\times 10^{12}$ ($7.03\times 10^{10}$)−	$1.4629\times 10^{12}$ ($1.33\times 10^{11}$)−	$5.1382\times 10^{12}$ ($6.14\times 10^{11}$)
MaF12	5	$2.8361\times 10^{8}$ ($1.13\times 10^{7}$)−	$2.5124\times 10^{8}$ ($1.05\times 10^{7}$)−	$3.2424\times 10^{8}$ ($1.25\times 10^{7}$)−	$2.4679\times 10^{8}$ ($5.15\times 10^{6}$)−	$6.6300\times 10^{8}$ ($9.77\times 10^{6}$)
	10	$4.7870\times 10^{11}$ ($2.63\times 10^{10}$)−	$2.9514\times 10^{11}$ ($1.82\times 10^{10}$)−	$5.6689\times 10^{11}$ ($3.99\times 10^{10}$)−	$3.6467\times 10^{11}$ ($1.21\times 10^{10}$)−	$1.7000\times 10^{12}$ ($4.06\times 10^{10}$)
	15	$3.3691\times 10^{13}$ ($2.15\times 10^{12}$)−	$1.1130\times 10^{13}$ ($1.20\times 10^{12}$)−	$3.2282\times 10^{13}$ ($3.95\times 10^{12}$)−	$1.8148\times 10^{13}$ ($1.51\times 10^{12}$)−	$9.7656\times 10^{13}$ ($5.11\times 10^{12}$)
MaF13	5	$1.6838\times 10^{12}$ ($2.83\times 10^{12}$)+	$5.1837\times 10^{10}$ ($2.56\times 10^{11}$)−	$2.3224\times 10^{10}$ ($1.09\times 10^{11}$)−	$2.9910\times 10^{9}$ ($1.00\times 10^{9}$)−	$1.0670\times 10^{12}$ ($5.84\times 10^{12}$)
	10	$2.3591\times 10^{17}$ ($4.48\times 10^{17}$)+	$2.0559\times 10^{18}$ ($6.80\times 10^{18}$)=	$4.2493\times 10^{10}$ ($1.56\times 10^{10}$)−	$1.7504\times 10^{14}$ ($6.47\times 10^{14}$)=	$8.5663\times 10^{13}$ ($3.40\times 10^{14}$)
	15	$4.4817\times 10^{19}$ ($7.71\times 10^{19}$)+	$3.2243\times 10^{23}$ ($7.60\times 10^{23}$)+	$7.7767\times 10^{12}$ ($2.42\times 10^{13}$)−	$3.6364\times 10^{15}$ ($1.38\times 10^{16}$)−	$1.2819\times 10^{19}$ ($5.13\times 10^{19}$)
MaF14	5	$3.9516\times 10^{8}$ ($2.27\times 10^{8}$)−	$1.1520\times 10^{8}$ ($5.74\times 10^{7}$)−	$5.3314\times 10^{7}$ ($2.79\times 10^{7}$)−	$1.7354\times 10^{9}$ ($1.52\times 10^{9}$)=	$2.9699\times 10^{9}$ ($4.14\times 10^{9}$)
	10	$2.9858\times 10^{11}$ ($3.32\times 10^{11}$)+	$3.4903\times 10^{10}$ ($3.02\times 10^{10}$)=	$7.6269\times 10^{10}$ ($4.54\times 10^{10}$)+	$3.0495\times 10^{10}$ ($3.54\times 10^{10}$)−	$4.9813\times 10^{10}$ ($3.35\times 10^{10}$)
	15	$4.6355\times 10^{13}$ ($4.55\times 10^{13}$)+	$2.0714\times 10^{12}$ ($1.66\times 10^{12}$)=	$1.0548\times 10^{13}$ ($5.15\times 10^{12}$)+	$4.3050\times 10^{13}$ ($1.06\times 10^{14}$)+	$4.2768\times 10^{12}$ ($6.82\times 10^{12}$)
MaF15	5	$4.5279\times 10^{8}$ ($5.90\times 10^{7}$)+	$6.8619\times 10^{7}$ ($1.23\times 10^{7}$)−	$3.5312\times 10^{7}$ ($5.80\times 10^{6}$)−	$2.9326\times 10^{7}$ ($7.29\times 10^{6}$)−	$1.5157\times 10^{8}$ ($9.04\times 10^{7}$)
	10	$8.7891\times 10^{11}$ ($2.84\times 10^{11}$)+	$1.5901\times 10^{10}$ ($4.25\times 10^{9}$)−	$8.7310\times 10^{10}$ ($1.06\times 10^{10}$)−	$9.5543\times 10^{9}$ ($2.82\times 10^{9}$)−	$1.0067\times 10^{11}$ ($1.99\times 10^{11}$)
	15	$9.7562\times 10^{13}$ ($4.37\times 10^{13}$)+	$9.4054\times 10^{11}$ ($5.66\times 10^{11}$)−	$3.8922\times 10^{12}$ ($7.43\times 10^{11}$)+	$2.1926\times 10^{11}$ ($6.98\times 10^{10}$)−	$2.2061\times 10^{12}$ ($5.53\times 10^{11}$)
+/−/=		14/27/4	6/36/3	5/37/3	5/35/5	−−−−−−

Note: Bold marks indicate the best-performing results.

**Table 4:** Comparison of IGD values of six algorithms on WFG test problems.

Problems	M	NSGAIII	MOEA/DD	1by1EA	SPEAR	RVEA	MaOEADS
WFG1	5	$6.4633\times 10^{-1}$ ($6.51\times 10^{-2}$)+	$7.4770\times 10^{-1}$ ($1.29\times 10^{-1}$)+	$7.5595\times 10^{-1}$ ($5.79\times 10^{-2}$)+	$6.7558\times 10^{-1}$ ($3.99\times 10^{-2}$)+	$5.8124\times 10^{-1}$ ($8.22\times 10^{-2}$)+	$8.4710\times 10^{-1}$ ($1.12\times 10^{-1}$)
	10	$1.5127\times 10^{0}$ ($1.09\times 10^{-1}$)+	$1.4350\times 10^{0}$ ($6.92\times 10^{-2}$)+	$1.6295\times 10^{0}$ ($8.66\times 10^{-2}$)=	$1.7542\times 10^{0}$ ($6.39\times 10^{-2}$)−	$1.1013\times 10^{0}$ ($6.19\times 10^{-2}$)+	$1.6346\times 10^{0}$ ($7.39\times 10^{-2}$)
	15	$2.1024\times 10^{0}$ ($1.02\times 10^{-1}$)+	$2.2105\times 10^{0}$ ($1.51\times 10^{-1}$)=	$2.2549\times 10^{0}$ ($1.06\times 10^{-1}$)=	$2.4561\times 10^{0}$ ($1.17\times 10^{-1}$)−	$1.8934\times 10^{0}$ ($8.74\times 10^{-2}$)+	$2.1960\times 10^{0}$ ($7.18\times 10^{-2}$)
WFG2	5	$3.8803\times 10^{-1}$ ($3.55\times 10^{-3}$)+	$4.7795\times 10^{-1}$ ($1.49\times 10^{-2}$)=	$6.6180\times 10^{-1}$ ($6.58\times 10^{-2}$)−	$4.0011\times 10^{-1}$ ($3.72\times 10^{-3}$)+	$3.9465\times 10^{-1}$ ($1.49\times 10^{-2}$)+	$5.0158\times 10^{-1}$ ($1.44\times 10^{-1}$)
	10	$1.2728\times 10^{0}$ ($1.42\times 10^{-1}$)=	$1.3226\times 10^{0}$ ($3.92\times 10^{-2}$)=	$1.7248\times 10^{0}$ ($8.17\times 10^{-2}$)−	$1.0965\times 10^{0}$ ($1.73\times 10^{-2}$)+	$1.1439\times 10^{0}$ ($4.48\times 10^{-2}$)+	$1.3734\times 10^{0}$ ($1.04\times 10^{-1}$)
	15	$1.5488\times 10^{0}$ ($4.63\times 10^{-2}$)+	$1.9522\times 10^{0}$ ($6.30\times 10^{-2}$)+	$2.2395\times 10^{0}$ ($8.80\times 10^{-2}$)−	$1.5550\times 10^{0}$ ($3.15\times 10^{-2}$)+	$1.6373\times 10^{0}$ ($9.51\times 10^{-2}$)+	$2.1271\times 10^{0}$ ($2.50\times 10^{-1}$)
WFG3	5	$4.8297\times 10^{-1}$ ($4.07\times 10^{-2}$)+	$6.2896\times 10^{-1}$ ($5.17\times 10^{-2}$)−	$1.3403\times 10^{0}$ ($1.12\times 10^{-1}$)−	$4.6479\times 10^{-1}$ ($7.07\times 10^{-2}$)+	$5.1447\times 10^{-1}$ ($3.95\times 10^{-2}$)=	$5.2335\times 10^{-1}$ ($3.57\times 10^{-2}$)
	10	$1.1622\times 10^{0}$ ($3.43\times 10^{-1}$)=	$2.7565\times 10^{0}$ ($1.36\times 10^{-1}$)−	$5.4942\times 10^{0}$ ($7.44\times 10^{-1}$)−	$2.0733\times 10^{0}$ ($2.35\times 10^{-1}$)−	$3.4513\times 10^{0}$ ($9.10\times 10^{-1}$)−	$1.0877\times 10^{0}$ ($1.74\times 10^{-1}$)
	15	$2.3081\times 10^{0}$ ($4.60\times 10^{-1}$)−	$5.9429\times 10^{0}$ ($3.37\times 10^{-1}$)−	$1.0027\times 10^{1}$ ($2.09\times 10^{0}$)−	$4.1699\times 10^{0}$ ($3.26\times 10^{-1}$)−	$6.1477\times 10^{0}$ ($1.54\times 10^{0}$)−	$1.3342\times 10^{0}$ ($1.51\times 10^{-1}$)
WFG4	5	$9.6444\times 10^{-1}$ ($2.86\times 10^{-3}$)−	$1.0525\times 10^{0}$ ($2.43\times 10^{-3}$)−	$1.4603\times 10^{0}$ ($1.24\times 10^{-1}$)−	$9.7538\times 10^{-1}$ ($3.95\times 10^{-3}$)−	$9.5988\times 10^{-1}$ ($1.64\times 10^{-3}$)−	$8.4458\times 10^{-1}$ ($4.67\times 10^{-3}$)
	10	$4.5129\times 10^{0}$ ($3.76\times 10^{-2}$)−	$6.2603\times 10^{0}$ ($1.85\times 10^{-1}$)−	$5.8334\times 10^{0}$ ($1.50\times 10^{-1}$)−	$4.5545\times 10^{0}$ ($1.63\times 10^{-2}$)−	$4.3788\times 10^{0}$ ($5.36\times 10^{-2}$)−	$3.7674\times 10^{0}$ ($9.69\times 10^{-2}$)
	15	$8.1639\times 10^{0}$ ($1.04\times 10^{-1}$)−	$9.1861\times 10^{0}$ ($2.64\times 10^{-1}$)−	$1.0698\times 10^{1}$ ($2.73\times 10^{-1}$)−	$8.5551\times 10^{0}$ ($1.14\times 10^{-1}$)−	$8.8103\times 10^{0}$ ($4.47\times 10^{-1}$)−	$7.6513\times 10^{0}$ ($2.18\times 10^{-1}$)
WFG5	5	$9.4632\times 10^{-1}$ ($3.16\times 10^{-3}$)−	$1.0341\times 10^{0}$ ($3.69\times 10^{-3}$)−	$1.4240\times 10^{0}$ ($1.16\times 10^{-1}$)-	$9.6416\times 10^{-1}$ ($4.86\times 10^{-3}$)−	$9.5221\times 10^{-1}$ ($1.21\times 10^{-3}$)−	$8.4068\times 10^{-1}$ ($5.95\times 10^{-3}$)
	10	$4.4617\times 10^{0}$ ($2.56\times 10^{-2}$)−	$6.3231\times 10^{0}$ ($1.35\times 10^{-1}$)−	$5.9291\times 10^{0}$ ($2.09\times 10^{-1}$)−	$4.5370\times 10^{0}$ ($1.38\times 10^{-2}$)−	$4.3830\times 10^{0}$ ($6.85\times 10^{-2}$)−	$3.6612\times 10^{0}$ ($2.26\times 10^{-2}$)
	15	$7.8952\times 10^{0}$ ($2.53\times 10^{-1}$)−	$9.4691\times 10^{0}$ ($1.05\times 10^{-1}$)−	$1.1154\times 10^{1}$ ($1.97\times 10^{-1}$)−	$8.7390\times 10^{0}$ ($3.70\times 10^{-2}$)−	$8.5338\times 10^{0}$ ($2.28\times 10^{-1}$)−	$7.1526\times 10^{0}$ ($2.42\times 10^{-1}$)
WFG6	5	$9.6803\times 10^{-1}$ ($3.89\times 10^{-3}$)−	$1.0471\times 10^{0}$ ($3.93\times 10^{-3}$)−	$1.7912\times 10^{0}$ ($1.01\times 10^{-1}$)−	$9.7883\times 10^{-1}$ ($5.61\times 10^{-3}$)−	$9.6504\times 10^{-1}$ ($2.17\times 10^{-3}$)−	$8.8326\times 10^{-1}$ ($9.64\times 10^{-3}$)
	10	$4.5839\times 10^{0}$ ($1.77\times 10^{-2}$)−	$6.1404\times 10^{0}$ ($1.61\times 10^{-1}$)−	$6.8068\times 10^{0}$ ($1.76\times 10^{-1}$)−	$4.6045\times 10^{0}$ ($1.85\times 10^{-2}$)−	$4.3851\times 10^{0}$ ($7.54\times 10^{-2}$)−	$4.0783\times 10^{0}$ ($6.22\times 10^{-1}$)
	15	$7.9870\times 10^{0}$ ($3.09\times 10^{-1}$)−	$1.0504\times 10^{1}$ ($3.10\times 10^{-1}$)−	$1.2767\times 10^{1}$ ($3.03\times 10^{-1}$)−	$8.7871\times 10^{0}$ ($3.64\times 10^{-2}$)−	$9.0782\times 10^{0}$ ($3.30\times 10^{-1}$)−	$7.4515\times 10^{0}$ ($5.85\times 10^{-1}$)
WFG7	5	$9.6426\times 10^{-1}$ ($2.04\times 10^{-3}$)−	$1.0626\times 10^{0}$ ($4.40\times 10^{-3}$)−	$1.9143\times 10^{0}$ ($1.44\times 10^{-1}$)−	$9.7174\times 10^{-1}$ ($2.82\times 10^{-3}$)−	$9.6430\times 10^{-1}$ ($1.97\times 10^{-3}$)−	$8.4367\times 10^{-1}$ ($6.11\times 10^{-3}$)
	10	$4.5223\times 10^{0}$ ($6.67\times 10^{-2}$)−	$5.3847\times 10^{0}$ ($2.00\times 10^{-1}$)−	$6.0856\times 10^{0}$ ($2.98\times 10^{-1}$)−	$4.5791\times 10^{0}$ ($2.61\times 10^{-2}$)−	$4.3077\times 10^{0}$ ($5.42\times 10^{-2}$)−	$3.6845\times 10^{0}$ ($1.73\times 10^{-2}$)
	15	$8.1571\times 10^{0}$ ($6.56\times 10^{-2}$)−	$8.4210\times 10^{0}$ ($5.68\times 10^{-1}$)−	$9.8908\times 10^{0}$ ($4.71\times 10^{-1}$)−	$8.7380\times 10^{0}$ ($8.30\times 10^{-2}$)−	$8.0012\times 10^{0}$ ($4.04\times 10^{-1}$)−	$7.3786\times 10^{0}$ ($1.05\times 10^{-1}$)
WFG8	5	$1.0012\times 10^{0}$ ($9.84\times 10^{-3}$)+	$1.0640\times 10^{0}$ ($3.83\times 10^{-3}$)−	$1.6039\times 10^{0}$ ($8.35\times 10^{-2}$)−	$1.0091\times 10^{0}$ ($7.04\times 10^{-3}$)+	$1.0047\times 10^{0}$ ($2.44\times 10^{-3}$)+	$1.0140\times 10^{0}$ ($7.00\times 10^{-3}$)
	10	$4.5879\times 10^{0}$ ($2.95\times 10^{-1}$)−	$5.6383\times 10^{0}$ ($3.41\times 10^{-1}$)−	$6.6225\times 10^{0}$ ($4.74\times 10^{-1}$)−	$4.7420\times 10^{0}$ ($5.09\times 10^{-2}$)−	$4.4048\times 10^{0}$ ($1.12\times 10^{-1}$)−	$4.3220\times 10^{0}$ ($1.01\times 10^{-1}$)
	15	$8.3752\times 10^{0}$ ($5.81\times 10^{-1}$)+	$1.0623\times 10^{1}$ ($3.65\times 10^{-1}$)−	$1.1101\times 10^{1}$ ($9.33\times 10^{-1}$)−	$8.8914\times 10^{0}$ ($5.30\times 10^{-2}$)+	$8.2328\times 10^{0}$ ($5.57\times 10^{-1}$)+	$9.1297\times 10^{0}$ ($1.24\times 10^{-1}$)
WFG9	5	$9.3306\times 10^{-1}$ ($5.68\times 10^{-3}$)−	$1.0374\times 10^{0}$ ($6.20\times 10^{-3}$)−	$1.3834\times 10^{0}$ ($1.29\times 10^{-1}$)−	$9.4986\times 10^{-1}$ ($1.34\times 10^{-2}$)−	$9.4202\times 10^{-1}$ ($3.64\times 10^{-3}$)−	$8.4771\times 10^{-1}$ ($1.06\times 10^{-2}$)
	10	$4.2991\times 10^{0}$ ($5.31\times 10^{-2}$)−	$5.7643\times 10^{0}$ ($2.69\times 10^{-1}$)−	$5.3871\times 10^{0}$ ($1.84\times 10^{-1}$)−	$4.5102\times 10^{0}$ ($2.56\times 10^{-2}$)−	$4.3134\times 10^{0}$ ($6.46\times 10^{-2}$)−	$3.7706\times 10^{0}$ ($6.76\times 10^{-2}$)
	15	$7.9697\times 10^{0}$ ($2.06\times 10^{-1}$)−	$8.8195\times 10^{0}$ ($2.90\times 10^{-1}$)−	$9.7966\times 10^{0}$ ($2.32\times 10^{-1}$)−	$8.7412\times 10^{0}$ ($5.33\times 10^{-2}$)−	$7.5711\times 10^{0}$ ($2.62\times 10^{-1}$)−	$0.2358\times 10^{0}$ ($1.39\times 10^{-1}$)
+/−/=		8/17/2	3/21/31	1/24/2	7/20/0	8/18/1	−−−−−−

Note: Bold marks indicate the best-performing results.

**Table 5:** Comparison of PD values of six algorithms on WFG test problems.

Problems	M	NSGAIII	MOEA/DD	1by1EA	SPEAR	RVEA	MaOEADS
WFG1	5	$1.1698\times 10^{8}$ ($7.23\times 10^{6}$)+	$1.0498\times 10^{8}$ ($9.20\times 10^{6}$)=	$8.7496\times 10^{7}$ ($1.21\times 10^{7}$)=	$1.0863\times 10^{8}$ ($7.97\times 10^{6}$)=	$1.0821\times 10^{8}$ ($1.18\times 10^{7}$)=	$9.8028\times 10^{7}$ ($2.35\times 10^{7}$)
	10	$8.7220\times 10^{10}$ ($6.70\times 10^{9}$)+	$4.8276\times 10^{10}$ ($5.47\times 10^{9}$)−	$6.8518\times 10^{10}$ ($1.14\times 10^{10}$)−	$6.3103\times 10^{10}$ ($9.06\times 10^{9}$)−	$5.9034\times 10^{10}$ ($7.24\times 10^{9}$)−	$7.7569\times 10^{10}$ ($1.26\times 10^{10}$)
	15	$2.9552\times 10^{12}$ ($3.40\times 10^{11}$)=	$2.2892\times 10^{12}$ ($2.35\times 10^{11}$)−	$2.7933\times 10^{12}$ ($5.33\times 10^{11}$)=	$2.1454\times 10^{12}$ ($2.17\times 10^{11}$)−	$1.5950\times 10^{12}$ ($3.26\times 10^{11}$)−	$2.7561\times 10^{12}$ ($5.97\times 10^{11}$)
WFG2	5	$1.0417\times 10^{8}$ ($4.30\times 10^{6}$)−	$8.2058\times 10^{7}$ ($2.79\times 10^{6}$)−	$8.9302\times 10^{7}$ ($4.10\times 10^{6}$)−	$9.0338\times 10^{7}$ ($3.38\times 10^{6}$)−	$9.8692\times 10^{7}$ ($3.74\times 10^{6}$)−	$1.5066\times 10^{8}$ ($1.79\times 10^{7}$)
	10	$8.0549\times 10^{10}$ ($1.89\times 10^{10}$)−	$4.8425\times 10^{10}$ ($3.95\times 10^{9}$)−	$7.0575\times 10^{10}$ ($2.51\times 10^{9}$)−	$7.6180\times 10^{10}$ ($3.51\times 10^{9}$)−	$5.7366\times 10^{10}$ ($3.09\times 10^{9}$)−	$1.3398\times 10^{11}$ ($8.26\times 10^{9}$)
	15	$3.4325\times 10^{12}$ ($2.68\times 10^{11}$)−	$9.8461\times 10^{11}$ ($1.48\times 10^{11}$)−	$2.6482\times 10^{12}$ ($1.17\times 10^{11}$)−	$3.1218\times 10^{12}$ ($2.24\times 10^{11}$)−	$2.3737\times 10^{12}$ ($3.89\times 10^{11}$)−	$5.1493\times 10^{12}$ ($3.22\times 10^{11}$)
WFG3	5	$2.0776\times 10^{8}$ ($8.32\times 10^{6}$)−	$1.4575\times 10^{8}$ ($7.26\times 10^{6}$)−	$2.2350\times 10^{8}$ ($7.10\times 10^{6}$)−	$9.3675\times 10^{7}$ ($9.95\times 10^{6}$)−	$1.6148\times 10^{8}$ ($1.81\times 10^{7}$)−	$3.1291\times 10^{8}$ ($1.10\times 10^{7}$)
	10	$2.5679\times 10^{11}$ ($2.36\times 10^{10}$)−	$1.0499\times 10^{11}$ ($6.69\times 10^{9}$)−	$2.5963\times 10^{11}$ ($1.32\times 10^{10}$)−	$7.5062\times 10^{10}$ ($6.88\times 10^{9}$)−	$1.6407\times 10^{11}$ ($1.75\times 10^{10}$)−	$5.3135\times 10^{11}$ ($3.54\times 10^{10}$)
	15	$1.0619\times 10^{13}$ ($2.44\times 10^{12}$)−	$5.1565\times 10^{12}$ ($3.98\times 10^{11}$)−	$9.9983\times 10^{12}$ ($9.46\times 10^{11}$)−	$4.1787\times 10^{12}$ ($8.34\times 10^{11}$)−	$8.7560\times 10^{12}$ ($2.02\times 10^{12}$)−	$2.1459\times 10^{13}$ ($2.01\times 10^{12}$)
WFG4	5	$2.1048\times 10^{8}$ ($6.17\times 10^{6}$)−	$1.8905\times 10^{8}$ ($4.49\times 10^{6}$)−	$2.5200\times 10^{8}$ ($1.23\times 10^{7}$)−	$1.9124\times 10^{8}$ ($7.59\times 10^{6}$)−	$2.0630\times 10^{8}$ ($7.16\times 10^{6}$)−	$5.6277\times 10^{8}$ ($1.42\times 10^{7}$)
	10	$2.9616\times 10^{11}$ ($2.95\times 10^{10}$)−	$1.4211\times 10^{11}$ ($9.49\times 10^{9}$)−	$2.7562\times 10^{11}$ ($1.53\times 10^{10}$)−	$2.4797\times 10^{11}$ ($1.33\times 10^{10}$)−	$1.9412\times 10^{11}$ ($1.49\times 10^{10}$)−	$1.1261\times 10^{12}$ ($7.25\times 10^{10}$)
	15	$2.3520\times 10^{13}$ ($2.24\times 10^{12}$)−	$8.4796\times 10^{12}$ ($6.41\times 10^{11}$)−	$9.8140\times 10^{12}$ ($7.24\times 10^{11}$)−	$1.6176\times 10^{13}$ ($1.77\times 10^{12}$)−	$1.0200\times 10^{13}$ ($5.39\times 10^{12}$)−	$5.9666\times 10^{13}$ ($4.48\times 10^{12}$)
WFG5	5	$2.2018\times 10^{8}$ ($8.09\times 10^{6}$)−	$1.8600\times 10^{8}$ ($5.66\times 10^{6}$)−	$2.5992\times 10^{8}$ ($9.43\times 10^{6}$)−	$1.6127\times 10^{8}$ ($5.90\times 10^{6}$)−	$1.8980\times 10^{8}$ ($5.41\times 10^{6}$)−	$5.8850\times 10^{8}$ ($1.33\times 10^{7}$)
	10	$3.7819\times 10^{11}$ ($1.57\times 10^{10}$)−	$1.8887\times 10^{11}$ ($1.21\times 10^{10}$)−	$3.3906\times 10^{11}$ ($1.69\times 10^{10}$)−	$2.7034\times 10^{11}$ ($1.24\times 10^{10}$)−	$2.2836\times 10^{11}$ ($8.23\times 10^{9}$)−	$1.3563\times 10^{12}$ ($2.97\times 10^{10}$)
	15	$3.0427\times 10^{13}$ ($2.10\times 10^{12}$)−	$4.8106\times 10^{12}$ ($4.73\times 10^{11}$)−	$1.4339\times 10^{13}$ ($7.43\times 10^{11}$)−	$1.1252\times 10^{13}$ ($5.24\times 10^{11}$)−	$1.0359\times 10^{13}$ ($6.24\times 10^{11}$)−	$7.4247\times 10^{13}$ ($7.89\times 10^{12}$)
WFG6	5	$1.9846\times 10^{8}$ ($7.92\times 10^{6}$)−	$1.7656\times 10^{8}$ ($1.01\times 10^{7}$)−	$2.2277\times 10^{8}$ ($1.44\times 10^{7}$)−	$1.8307\times 10^{8}$ ($1.30\times 10^{7}$)−	$1.8611\times 10^{8}$ ($7.21\times 10^{6}$)−	$5.7775\times 10^{8}$ ($1.90\times 10^{7}$)
	10	$2.7307\times 10^{11}$ ($1.42\times 10^{10}$)−	$1.6171\times 10^{11}$ ($1.01\times 10^{10}$)−	$2.3104\times 10^{11}$ ($1.39\times 10^{10}$)−	$2.2149\times 10^{11}$ ($1.23\times 10^{10}$)−	$2.0821\times 10^{11}$ ($9.68\times 10^{9}$)−	$1.1482\times 10^{12}$ ($1.57\times 10^{11}$)
	15	$2.5428\times 10^{13}$ ($1.56\times 10^{12}$)−	$4.2348\times 10^{12}$ ($4.03\times 10^{11}$)−	$8.8174\times 10^{12}$ ($7.38\times 10^{11}$)−	$1.0614\times 10^{13}$ ($5.54\times 10^{11}$)−	$8.5766\times 10^{12}$ ($5.11\times 10^{11}$)−	$6.5029\times 10^{13}$ ($8.12\times 10^{12}$)
WFG7	5	$2.1256\times 10^{8}$ ($7.49\times 10^{6}$)−	$2.0780\times 10^{8}$ ($7.84\times 10^{6}$)−	$2.2249\times 10^{8}$ ($1.77\times 10^{7}$)−	$2.1776\times 10^{8}$ ($6.16\times 10^{6}$)−	$2.0790\times 10^{8}$ ($5.42\times 10^{6}$)−	$6.0355\times 10^{8}$ ($1.60\times 10^{7}$)
	10	$3.8882\times 10^{11}$ ($1.76\times 10^{10}$)−	$2.5269\times 10^{11}$ ($1.07\times 10^{10}$)−	$3.3607\times 10^{11}$ ($2.07\times 10^{10}$)−	$4.2304\times 10^{11}$ ($1.42\times 10^{10}$)−	$2.6579\times 10^{11}$ ($1.31\times 10^{10}$)−	$1.3589\times 10^{12}$ ($3.25\times 10^{10}$)
	15	$3.4182\times 10^{13}$ ($2.65\times 10^{12}$)−	$1.1550\times 10^{13}$ ($1.03\times 10^{12}$)−	$2.7436\times 10^{13}$ ($4.63\times 10^{12}$)−	$1.7104\times 10^{13}$ ($7.96\times 10^{11}$)−	$2.2836\times 10^{13}$ ($2.25\times 10^{12}$)−	$6.8444\times 10^{13}$ ($4.12\times 10^{12}$)
WFG8	5	$2.8928\times 10^{8}$ ($9.24\times 10^{6}$)−	$2.0857\times 10^{8}$ ($6.03\times 10^{6}$)−	$2.7642\times 10^{8}$ ($1.50\times 10^{7}$)−	$2.5004\times 10^{8}$ ($8.16\times 10^{6}$)−	$2.7011\times 10^{8}$ ($9.01\times 10^{6}$)−	$6.5212\times 10^{8}$ ($1.35\times 10^{7}$)
	10	$4.2663\times 10^{11}$ ($6.03\times 10^{10}$)−	$1.7327\times 10^{11}$ ($1.67\times 10^{10}$)−	$2.5967\times 10^{11}$ ($3.51\times 10^{10}$)−	$3.8069\times 10^{11}$ ($1.92\times 10^{10}$)−	$2.3532\times 10^{11}$ ($2.48\times 10^{10}$)−	$1.3206\times 10^{12}$ ($7.37\times 10^{10}$)
	15	$2.7028\times 10^{13}$ ($2.55\times 10^{12}$)−	$5.3345\times 10^{12}$ ($1.74\times 10^{12}$)−	$1.5807\times 10^{13}$ ($4.38\times 10^{12}$)−	$1.1487\times 10^{13}$ ($7.52\times 10^{11}$)−	$1.0916\times 10^{13}$ ($2.32\times 10^{12}$)−	$5.0140\times 10^{13}$ ($4.27\times 10^{12}$)
WFG9	5	$3.3322\times 10^{8}$ ($1.10\times 10^{7}$)−	$2.7580\times 10^{8}$ ($9.91\times 10^{6}$)−	$3.2949\times 10^{8}$ ($1.08\times 10^{7}$)−	$3.3025\times 10^{8}$ ($9.21\times 10^{6}$)−	$2.9230\times 10^{8}$ ($7.96\times 10^{6}$)−	$6.6656\times 10^{8}$ ($1.07\times 10^{7}$)
	10	$6.1450\times 10^{11}$ ($3.79\times 10^{10}$)−	$3.3937\times 10^{11}$ ($2.46\times 10^{10}$)−	$6.2123\times 10^{11}$ ($3.19\times 10^{10}$)−	$5.8324\times 10^{11}$ ($2.77\times 10^{10}$)−	$4.2975\times 10^{11}$ ($2.22\times 10^{10}$)−	$1.7008\times 10^{12}$$1.03\times 10^{11}$)
	15	$4.1330\times 10^{13}$ ($3.18\times 10^{12}$)−	$1.5652\times 10^{13}$ ($1.72\times 10^{12}$)−	$3.8363\times 10^{13}$ ($3.63\times 10^{12}$)−	$2.2601\times 10^{13}$ ($1.41\times 10^{12}$)−	$2.4666\times 10^{13}$ ($2.94\times 10^{12}$)−	$9.7837\times 10^{13}$ ($5.15\times 10^{12}$)
+/−/=		2/24/1	0/26/1	0/25/2	0/26/1	0/26/1	−−−−−−

Note: Bold marks indicate the best-performing results.

**Table 6:** Comparison of IGD values of four algorithms on DTLZ test problems.

Problems	M	BiGE	KnEA	Two_Arch2	MaOEADS
DTLZ1	5	$1.0159\times 10^{-1}$ ($1.87\times 10^{-2}$) −	$1.4675\times 10^{-1}$ ($7.51\times 10^{-2}$) −	$5.2960\times 10^{-2}$ ($7.29\times 10^{-4}$) =	$5.6934\times 10^{-2}$ ($7.30\times 10^{-3}$)
	10	$5.0422\times 10^{-1}$ ($2.11\times 10^{-1}$) −	$5.2582\times 10^{0}$ ($3.95\times 10^{0}$) −	$1.0576\times 10^{-1}$ ($1.28\times 10^{-3}$) +	$1.2236\times 10^{-1}$ ($1.47\times 10^{-2}$)
	15	$8.0216\times 10^{-1}$ ($3.74\times 10^{-1}$) −	$2.8731\times 10^{0}$ ($2.18\times 10^{0}$) −	$1.4030\times 10^{-1}$ ($2.61\times 10^{-3}$) =	$1.5616\times 10^{-1}$ ($6.09\times 10^{-2}$)
DTLZ2	5	$2.0888\times 10^{-1}$ ($3.05\times 10^{-3}$) −	$1.7292\times 10^{-1}$ ($2.33\times 10^{-3}$) −	$1.7280\times 10^{-1}$ ($1.94\times 10^{-3}$) −	$1.4821\times 10^{-1}$ ($1.46\times 10^{-3}$)
	10	$4.5028\times 10^{-1}$ ($5.72\times 10^{-3}$) −	$4.7440\times 10^{-1}$ ($9.65\times 10^{-2}$) −	$4.2212\times 10^{-1}$ ($2.65\times 10^{-3}$) −	$3.8524\times 10^{-1}$ ($5.50\times 10^{-3}$)
	15	$5.7849\times 10^{-1}$ ($6.96\times 10^{-3}$) −	$6.4483\times 10^{-1}$ ($1.45\times 10^{-1}$) −	$6.2168\times 10^{-1}$ ($9.58\times 10^{-3}$) −	$5.2766\times 10^{-1}$ ($7.28\times 10^{-3}$)
DTLZ3	5	$5.8813\times 10^{-1}$ ($2.53\times 10^{-1}$) −	$3.9517\times 10^{-1}$ ($1.64\times 10^{-1}$) −	$2.0178\times 10^{-1}$ ($1.77\times 10^{-2}$) −	$1.7687\times 10^{-1}$ ($2.24\times 10^{-2}$)
	10	$2.1921\times 10^{1}$ ($6.63\times 10^{0}$) −	$3.6317\times 10^{2}$ ($7.40\times 10^{1}$) −	$5.4771\times 10^{-1}$ ($5.66\times 10^{-2}$) +	$1.0025\times 10^{0}$ ($4.93\times 10^{-1}$)
	15	$3.2595\times 10^{1}$ ($1.30\times 10^{1}$) −	$7.4939\times 10^{2}$ ($1.21\times 10^{2}$) −	$3.1432\times 10^{0}$ ($2.25\times 10^{0}$) −	$1.3577\times 10^{0}$ ($5.58\times 10^{-1}$)
DTLZ4	5	$2.0948\times 10^{-1}$ ($4.91\times 10^{-3}$) +	$1.7147\times 10^{-1}$ ($5.36\times 10^{-3}$) +	$1.7499\times 10^{-1}$ ($2.65\times 10^{-3}$) +	$8.9514\times 10^{-1}$ ($3.45\times 10^{-2}$)
	10	$6.4786\times 10^{-1}$ ($2.16\times 10^{-2}$) −	$4.4757\times 10^{-1}$ ($3.97\times 10^{-3}$) −	$4.1765\times 10^{-1}$ ($2.68\times 10^{-3}$) −	$3.8072\times 10^{-1}$ ($2.42\times 10^{-3}$)
	15	$7.9061\times 10^{-1}$ ($8.73\times 10^{-3}$) −	$5.6598\times 10^{-1}$ ($7.26\times 10^{-3}$) −	$5.6315\times 10^{-1}$ ($3.68\times 10^{-3}$) −	$5.1872\times 10^{-1}$ ($2.38\times 10^{-3}$)
DTLZ5	5	$9.8499\times 10^{-2}$ ($1.36\times 10^{-2}$) −	$1.5473\times 10^{-1}$ ($3.62\times 10^{-2}$) −	$5.9792\times 10^{-2}$ ($9.37\times 10^{-3}$) =	$6.3489\times 10^{-2}$ ($9.84\times 10^{-3}$)
	10	$3.2611\times 10^{-1}$ ($8.34\times 10^{-2}$) −	$3.0687\times 10^{-1}$ ($5.77\times 10^{-2}$) −	$1.6977\times 10^{-1}$ ($3.37\times 10^{-2}$) −	$1.1390\times 10^{-1}$ ($2.48\times 10^{-2}$)
	15	$4.0924\times 10^{-1}$ ($3.64\times 10^{-2}$) −	$5.0398\times 10^{-1}$ ($2.43\times 10^{-1}$) −	$3.5927\times 10^{-1}$ ($1.23\times 10^{-1}$) −	$1.2167\times 10^{-1}$ ($2.45\times 10^{-2}$)
DTLZ6	5	$6.5350\times 10^{-1}$ ($7.64\times 10^{-2}$) −	$2.5323\times 10^{-1}$ ($8.99\times 10^{-2}$) −	$8.5504\times 10^{-2}$ ($1.45\times 10^{-2}$) −	$5.4738\times 10^{-2}$ ($1.08\times 10^{-2}$)
	10	$6.5240\times 10^{-1}$ ($7.46\times 10^{-2}$) −	$2.3265\times 10^{0}$ ($5.92\times 10^{-1}$) −	$3.6897\times 10^{-1}$ ($1.36\times 10^{-1}$) −	$1.9888\times 10^{-1}$ ($6.62\times 10^{-2}$)
	15	$7.2410\times 10^{-1}$ ($1.66\times 10^{-1}$) −	$2.3771\times 10^{0}$ ($4.87\times 10^{-1}$) −	$3.1099\times 10^{0}$ ($6.89\times 10^{-1}$) −	$2.2409\times 10^{-1}$ ($6.48\times 10^{-2}$)
DTLZ7	5	$3.8680\times 10^{-1}$ ($1.17\times 10^{-1}$) +	$2.4199\times 10^{-1}$ ($6.93\times 10^{-3}$) +	$2.5333\times 10^{-1}$ ($6.32\times 10^{-2}$) +	$1.4964\times 10^{0}$ ($8.52\times 10^{-3}$)
	10	$3.4717\times 10^{0}$ ($4.30\times 10^{-1}$) =	$8.7240\times 10^{-1}$ ($8.63\times 10^{-3}$) +	$9.3805\times 10^{-1}$ ($3.02\times 10^{-2}$) +	$3.5455\times 10^{0}$ ($1.36\times 10^{-1}$)
	15	$1.0394\times 10^{1}$ ($4.39\times 10^{-1}$) −	$4.9742\times 10^{0}$ ($7.79\times 10^{-1}$) +	$6.8648\times 10^{0}$ ($1.22\times 10^{0}$) +	$7.7659\times 10^{0}$ ($3.40\times 10^{-1}$)
+/−/=		2/18/1	4/17/0	6/12/3	−−−−−−

Note: Bold marks indicate the best-performing results.

**Table 7:** The IGD values of NSGA-III and NSGA-III-PC on the DTLZ test problem.

Problem	M = 5		M = 10		M = 15	
	NSGAIII	NSGAIII-PC	NSGAIII	NSGAIII-PC	NSGAIII	NSGAIII-PC
DTLZ1	$6.7832\times 10^{-1}$ ($3.19\times 10^{-1}$)−	$7.8151\times 10^{-2}$ ($1.55\times 10^{-2}$)	$4.1924\times 10^{0}$ ($2.09\times 10^{0}$)−	$7.9292\times 10^{-2}$ ($9.07\times 10^{-2}$)	$3.9094\times 10^{0}$ ($1.55\times 10^{0}$)−	$5.2415\times 10^{-2}$ ($5.02\times 10^{-2}$)
DTLZ2	$2.1379\times 10^{-1}$ ($5.30\times 10^{-4}$)−	$1.1978\times 10^{-1}$ ($8.01\times 10^{-3}$)	$5.2921\times 10^{-1}$ ($6.93\times 10^{-2}$)−	$8.7973\times 10^{-2}$ ($1.53\times 10^{-1}$)	$6.9242\times 10^{-1}$ ($4.91\times 10^{-2}$)−	$3.0851\times 10^{-1}$ ($1.11\times 10^{-1}$)
DTLZ3	$2.5655\times 10^{0}$ ($2.04\times 10^{0}$)−	$3.3894\times 10^{-1}$ ($4.86\times 10^{-2}$)	$2.2668\times 10^{1}$ ($7.98\times 10^{0}$)−	$1.3876\times 10^{-1}$ ($1.89\times 10^{-1}$)	$2.4893\times 10^{1}$ ($7.91\times 10^{0}$)−	$3.0549\times 10^{-1}$ ($2.19\times 10^{-1}$)
DTLZ4	$3.1866\times 10^{-1}$ ($1.37\times 10^{-1}$) −	$1.2052\times 10^{-1}$ ($1.44\times 10^{-2}$)	$4.8177\times 10^{-1}$ ($2.22\times 10^{-2}$)−	$8.4755\times 10^{-2}$ ($1.46\times 10^{-1}$)	$6.5073\times 10^{-1}$ ($7.70\times 10^{-2}$)−	$2.4466\times 10^{-1}$ ($1.39\times 10^{-1}$)
DTLZ5	$1.1653\times 10^{-1}$ ($5.92\times 10^{-2}$)+	$6.3769\times 10^{-1}$ ($1.93\times 10^{-3}$)	$1.6189\times 10^{-1}$ ($6.26\times 10^{-2}$)+	$1.0631\times 10^{0}$ ($1.47\times 10^{-2}$)	$2.3604\times 10^{-1}$ ($6.35\times 10^{-2}$)+	$1.1660\times 10^{0}$ ($9.52\times 10^{-3}$)
DTLZ6	$3.6662\times 10^{-1}$ ($3.63\times 10^{-1}$)−	$4.8239\times 10^{2}$ ($5.17\times 10^{-1}$)	$2.9241\times 10^{0}$ ($3.46\times 10^{-1}$)−	$1.9805\times 10^{0}$ ($6.59\times 10^{-1}$)	$2.9871\times 10^{0}$ ($5.53\times 10^{-1}$)−	$2.6509\times 10^{0}$ ($9.45\times 10^{-1}$)
DTLZ7	$4.1341\times 10^{-1}$ ($1.23\times 10^{-1}$)+	$3.2607\times 10^{0}$ ($4.18\times 10^{-2}$)	$4.2123\times 10^{0}$ ($1.00\times 10^{0}$)+	$1.0811\times 10^{1}$ ($1.46\times 10^{0}$)	$1.1936\times 10^{1}$ ($2.65\times 10^{0}$)+	$1.8970\times 10^{1}$ ($1.89\times 10^{0}$)
+/−/=	2/5/0	−−−−−−	2/5/0	−−−−−−	2/5/0	−−−−−−

Note: Bold marks indicate the best-performing results.

**Table 8:** The PD values of D1 and D2 on the DTLZ test problem.

Problem	M = 5		M = 10		M = 15	
	D1	D2	D1	D2	D1	D2
DTLZ1	$8.5212\times 10^{6}$ ($1.24\times 10^{7}$)−	$1.2628\times 10^{7}$ ($3.82\times 10^{7}$)	$1.7961\times 10^{7}$ ($2.22\times 10^{7}$)−	$2.7675\times 10^{7}$ ($4.85\times 10^{7}$)	$4.4747\times 10^{8}$ ($1.20\times 10^{9}$)+	$3.1625\times 10^{7}$ ($1.20\times 10^{9}$)
DTLZ2	$4.6448\times 10^{4}$ ($1.02\times 10^{5}$)−	$1.8988\times 10^{5}$ ($2.83\times 10^{5}$)	$1.9157\times 10^{7}$ ($1.96\times 10^{7}$)−	$3.3295\times 10^{7}$ ($4.23\times 10^{7}$)	$5.7880\times 10^{7}$ ($8.03\times 10^{7}$)−	$3.2982\times 10^{8}$ ($6.01\times 10^{8}$)
DTLZ3	$1.7157\times 10^{8}$ ($2.18\times 10^{8}$)−	$3.8958\times 10^{8}$ ($3.57\times 10^{8}$)	$3.2317\times 10^{9}$ ($6.83\times 10^{9}$)−	$3.4134\times 10^{9}$ ($3.88\times 10^{9}$)	$1.3651\times 10^{9}$ ($2.62\times 10^{9}$)−	$2.2978\times 10^{10}$ ($6.86\times 10^{10}$)
DTLZ4	$1.6642\times 10^{1}$ ($5.12\times 10^{1}$)+	$2.6147\times 10^{-1}$ ($2.13\times 10^{-1}$)	$1.1560\times 10^{0}$ ($8.73\times 10^{-1}$)−	$1.5995\times 10^{0}$ ($2.96\times 10^{0}$)	$3.5229\times 10^{2}$ ($7.78\times 10^{2}$)+	$2.9157\times 10^{2}$$(5.74\times 10^{2}$)
DTLZ5	$4.3082\times 10^{5}$ ($5.25\times 10^{5}$)−	$4.7445\times 10^{5}$ ($4.52\times 10^{5}$)	$2.7916\times 10^{8}$ ($1.90\times 10^{8}$)−	$3.6615\times 10^{8}$ ($2.31\times 10^{8}$)	$4.9313\times 10^{9}$ ($6.02\times 10^{9}$)−	$5.2796\times 10^{9}$ ($5.25\times 10^{9}$)
DTLZ6	$3.9990\times 10^{5}$ ($6.80\times 10^{5}$)+	$1.8958\times 10^{5}$ ($2.22\times 10^{5}$)	$1.3138\times 10^{7}$ ($1.46\times 10^{7}$)−	$1.6605\times 10^{7}$ ($2.75\times 10^{7}$)	$1.0316\times 10^{8}$ ($8.44\times 10^{7}$)−	$1.5215\times 10^{8}$ ($2.40\times 10^{8}$)
DTLZ7	$4.5275\times 10^{6}$ ($1.37\times 10^{6}$)−	$6.3918\times 10^{6}$ ($3.85\times 10^{6}$)	$9.7971\times 10^{9}$ ($1.73\times 10^{9}$)−	$1.0070\times 10^{10}$ ($1.33\times 10^{9}$)	$7.0671\times 10^{11}$ ($1.08\times 10^{11}$)−	$7.1353\times 10^{11}$ ($8.07\times 10^{10}$)
+/−/=	2/5/0	−−−−−−	0/7/0	−−−−−−	2/5/0	−−−−−−

Note: Bold marks indicate the best-performing results.

## Data Availability

The article contains the data which are also available from the corresponding authors upon reasonable request.
